# TOX correlates with prognosis, immune infiltration, and T cells exhaustion in lung adenocarcinoma

**DOI:** 10.1002/cam4.3324

**Published:** 2020-07-23

**Authors:** Longbin Guo, Xuanzi Li, Rongping Liu, Yulei Chen, Chen Ren, Shasha Du

**Affiliations:** ^1^ Department of Radiation Oncology Nanfang Hospital Southern Medical University Guangzhou China

**Keywords:** immune infiltration, immunotherapy, lung adenocarcinoma, prognosis, T cells exhaustion, TOX

## Abstract

**Background:**

Thymocyte selection‐associated high mobility group box (TOX) plays a crucial role on the development of innate immunity and tumor microenvironment. This study aims to explore the prognostic potential of TOX and comprehensively analyze the correlations between TOX, immune infiltration, and T cells function in diverse cancers particularly lung adenocarcinoma (LUAD).

**Methods:**

TIMER was used to analyze TOX expression in different cancers. Potential prognostic value of TOX was evaluated by the PrognoScan, Kaplan‐Meier Plotter, and GEPIA2. The relationships between TOX, immune infiltration, and related gene marker sets were analyzed by TIMER and GEPIA2. Single‐cell RNA‐seq for T cells in LUAD was analyzed to further investigate the correlations between TOX expression and different T cells populations.

**Results:**

TOX downregulates in most of the cancer types and correlates with poor prognosis in LUAD. TOX shows significant impacts on survival of LUAD with early stage, ever‐smoking, or low‐TMB status. Increased TOX expression positively correlates with high immune infiltration levels in most of the immune cells and functional T cells including exhausted T cells. Moreover, multiple key genes of exhausted T cells comprising PD‐1, TIM‐3, TIGHT, and CXCL13 have remarkable interaction with TOX. Specifically, TOX is observed with high enrichment in exhausted CD4^+^ and CD8^+^ T cells populations in single‐cell RNA‐seq analysis for LUAD.

**Conclusion:**

TOX is a prognosis‐related biomarker for multiple cancer types especially LUAD. Increased TOX expression significantly increase immune infiltration levels in most of the immune cells comprising CD8^+^ T cells, CD4^+^ T cells, mast cells, and functional T cells. Moreover, we verified that TOX highly correlates with exhausted T cells and is probable a critical regulator promoted T cells exhaustion in LUAD. Detection of TOX expression could help to predict prognosis and regulating TOX expression in exhausted T cells may offer a novel strategy in maximizing immunotherapy efficacy for LUAD.

## Graphical abstract

In this study, we identified TOX is a prognosis‐related biomarker for multiple cancer types especially lung adenocarcinoma and correlate with immune infiltration levels in most of immune cells and functional T cells. Meanwhile, TOX is observed with high enrichment in exhausted CD4^+^ and CD8^+^ T cells populations in single‐cell RNA‐seq analysis. Our findings suggested that detecting expression level of TOX may help to predict prognosis and regulating TOX expression in exhausted T cells may provide a potential strategy in maximizing immunotherapy efficacy for lung adenocarcinoma patients.

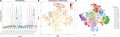

## 1 INTRODUCTION

Lung cancer remains a global public health problem that leads cause of cancer‐related mortality^1^. Non‐small cell lung carcinoma (NSCLC) including adenocarcinoma and squamous cell carcinoma comprises nearly 80%‐85% of all lung cancers.^1^, ^2^ Despite comprehensive therapy comprising surgical resection, chemotherapy and radiotherapy have improved clinical outcome in NSCLC, the 5‐year survival rate is still less than 20%.^1^, ^2^ Specific targeted therapies like tyrosine kinase inhibitors (TKIs) confer significant survival benefit in a minority of NSCLC patients with EGFR‐mutant, ALK‐rearranged, ROS1‐rearranged, or BRAF (V600E)‐mutant.^3^, ^4^, ^5^, ^6^, ^7^, ^8^, ^9^, ^10^ Over the last decade, immune checkpoint inhibitors (ICIs), particularly inhibitors of the anti‐programed cell death 1 (PD‐1) and anti‐programed cell death 1 ligand 1 (PD‐L1) axis, have demonstrated exceptional therapeutic landscape in NSCLC.^11^, ^12^, ^13^, ^14^ Some biomarkers, such as the PD‐L1 expression, tumor‐infiltration lymphocytes (TILs), TP53, and KRAS mutation status and tumor mutation burden (TMB), were reported for their predictive value for clinical responses in ICIs therapy.^15^, ^16^, ^17^ However, more novel effective biomarkers for immunotherapy response prediction or enhancements are necessary to explore.

TOX (thymocyte selection‐associated high mobility group box) was originally identified based on its upregulation during thymocyte differentiation and is expressed at specific stages of T cell development in the thymus.^18^ Subsequent researches demonstrated that TOX is an important DNA‐binding factor regulated development of various aspects of lymphocytes not just T cells.^19^, ^20^ Recently, TOX was revealed its crucial role in tumor‐specific T cell differentiation and CD8^+^ T cell exhaustion, highlighting a potential biomarker for response prediction or enhancement of cancer immunotherapy.^21^, ^22^ However, the correlations between TOX expression, prognosis, and immune infiltration in different cancers remain unclear.

This study comprehensively analyzed TOX expression and its prognostic value in various kinds of cancers using multiple databases including Tumor Immune Estimation Resource (TIMER), PrognoScan, Gene Expression Profiling Interactive Analysis 2 (GEPIA2), and Kaplan‐Meier plotter. The relationships between TOX expression and immune infiltration in different cancers were investigated via TIMER and GEPIA2. Moreover, single‐cell RNA‐seq for T cells in lung adenocarcinoma was acquired and analyzed in an open database to further explore the correlations between TOX expression and different T cells populations. We found that TOX is a potential prognosis‐related biomarker in LUAD and provided novel direction to understand the interactions between TOX expression, tumor infiltration, and T cells exhaustion.

## 2 METHODS

### 2.1 TIMER database analysis

TIMER (Tumor Immune Estimation Resource, cistrome.shinyapps.io/timer) is a user‐friendly web interface offering a comprehensive computational tool for oncology researchers to dynamic explore and visualize tumor immunologic and genomics data.^23^ The database providers analyzed gene expression data including 10 897 samples across 32 cancer types from The Cancer Genome Atlas (TCGA) to estimate the abundance of six tumor‐infiltrating immune cells (TIICs) subsets, including B cells, CD4^+^ T cells, CD8^+^ T cells, macrophages, neutrophils, and dendritic cells. Constrained least squares fitting was applied on the expression of selected genes, which negatively correlated with tumor purity for each cancer type,^24^ to predict the abundance of six TIIC subsets. In this work, we used “Diff Exp module” and “Gene module” to analyze TOX expression in various types of cancer and the correlations between TOX expression and the abundance of six TIIC subsets. Statistical significance of differential TOX expression was evaluated using Wilcoxon test. The correlations of TOX expression with immune infiltration were evaluated by purity‐corrected partial Spearman's correlation and statistical significance. “SCNA module” was used to compare tumor‐infiltration levels among tumors with different somatic copy number alterations for TOX. This module is defined by GISTIC 2.0, including deep deletion (−2), arm‐level deletion (−1), diploid/normal (0), arm‐level gain (1), and high amplification (2).^25^ Moreover, correlations between TOX expression and gene markers of tumor‐infiltration immune cells were investigated via “Correlation module.” The gene markers of tumor‐infiltrating immune cells (TIICs) were referenced previous studies and included markers of B cells, T cells, CD8^+^ T cells, effector T cells, effector memory T cells, central memory T cells, resident memory T cells, exhausted T cells, resting Treg cells, effector Treg cells, T‐helper 1 (Th1), macrophages, neutrophils, dendritic cells, natural killer cells (NK cells), and mast cells.^26^, ^27^, ^28^, ^29^ The module drew the expression scatterplots between TOX in a given cancer type, together with the Spearman's correlation and estimated statistical significance. Gene expression level was presented as log2 RSEM (RNA‐Seq by Expectation Maximization).

### 2.2 PrognoScan database analysis

PrognoScan database (http://dna00.bio.kyutech.ac.jp/PrognoScan/) is a large collection of publicly available cancer microarray datasets and a tool for assessing the biological relationships between gene expression and prognosis, providing a convenient platform to evaluate potential tumor markers and therapeutic targets.^30^ We used PrognoScan to investigate the association between TOX expression and survival in different types of cancers. COX *P*‐value, hazard ratio (HR) with 95% confidence intervals were calculated and displayed to evaluate the prognostic value of TOX.

### 2.3 Kaplan‐Meier plotter database analysis

Kaplan‐Meier Plotter database (http://kmplot.com/) is an online tool to rapidly access the effect of gene expression on survival in 21 cancer types, together with four large datasets including breast (n = 6234), ovarian (n = 2190), lung (n = 3452), and gastric (n = 1440) cancer.^31^ We used this tool to evaluate the correlations between TOX expression and survival in above four cancer types datasets. Further research about TOX expression in various subtypes of LUAD was performed via lung cancer dataset and pan‐cancer dataset. The log rank *P*‐value, HR (95% CI), and survival curves were also calculated and displayed.

### 2.4 GEPIA2 database analysis

GEPIA2 (Gene Expression Profiling Interactive Analysis 2) database (http://gepia2.cancer‐pku.cn/) is a web‐based tool for cancer and normal gene expression and interactive analysis based on TCGA and GTEx (Genotype‐Tissue Expression) data, providing customizable functions including differential expression analysis, profiling plotting, correlation analysis, patient survival analysis, similar gene detection and dimensionality reduction analysis.^32^ The relationships between TOX expression and survival in various types of cancers of TCGA were analyzed via “survival analysis.” Additionally, the correlations between TOX and gene markers of tumor‐infiltration immune cells were also performed using Spearman's correlation coefficient in “correlation analysis.” The tumor and normal tissue datasets were used for analysis.

### 2.5 Single‐cell analysis for T cells in LUAD

The single‐cell RNA‐seq for T cells in LUAD were acquired and analyzed in an online open database (http://lung.cancer‐pku.cn/) included 12 346 T cells from 14 treatment‐naïve non‐small cell lung cancer patients.^29^ The database applied unsupervised clustering based on t‐SNE+densityClust^33^ and identified 16 main clusters, including seven for CD8^+^ T cells (C1‐LEF1‐naïve CD8, C2‐CD28, C3‐CX3CR1‐effector CD8, C4‐GZMK, C5‐ZNF683‐tissue resident memory CD8, C6‐LAYN‐exhausted CD8, C7‐SLC4A10‐MAIT), seven for conventional CD4^+^ T cells (C1‐CCR7‐naïve CD4, C2‐ANXA1‐central memory CD4 in blood, C3‐GNLY‐effector CD4, C4‐CD69, C5‐EOMES, C6‐GZMA, and C7‐CXCL13‐exhausted CD4 of CD4 clusters), and two for regulatory T cells (C8‐FOXP3‐resting Tregs and C9‐CTLA4‐suppressive Tregs of CD4 clusters). LUAD patients (P0616A, P0616P, P0617, P0619, P0729, P1010, P1118, P1120, P1202, P1208, and P1219) were selected and the expression of TOX was normalized. Boxplot and t‐SNE plot for expression levels of TOX were generated to explore the correlations between TOX and different T cells populations in LUAD.

### 2.6 Statistical analysis

Distributions of TOX expression levels were displayed using box plots in TIMER, with statistical significance of differential expression evaluated using Wilcoxon test. Survival curves were generated from Kaplan‐Meier Plotter and GEPIA2 with HR and *P*‐value or Cox *P*‐value using log‐rank test. The infiltration level for each SCNA category is compared with the normal using two‐sided Wilcoxon rank‐sum test. The correlations between TOX expression and other gene or immune infiltration level in certain cancer type were evaluated by Spearman's correlation and statistical significance. Cutoff point was generally set in median unless otherwise specified. *P* ≤ .05 was considered statistically significant.

## 3 RESULTS

### 3.1 Expression level of TOX is downregulated in multiple cancer types

**Figure 1:**
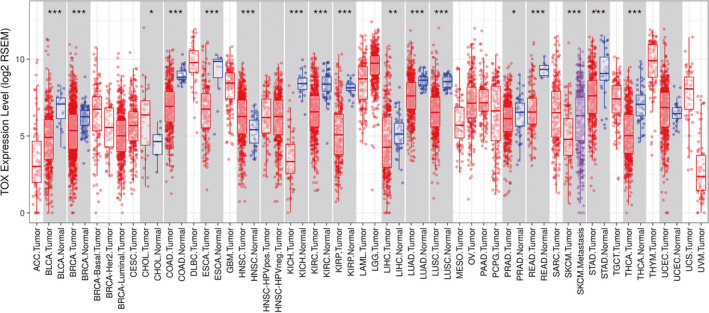
The expression levels of TOX in different cancer types from TCGA project demonstrated by TIMER. *P*‐value significant codes: 0 ≤ *** < .001 ≤ ** < .01 ≤ * < .05

In order to preliminarily evaluate the role of TOX in tumorigenesis, we analyzed the different expression levels of TOX between tumor and adjacent normal tissues in all TCGA tumors (Figure 1). Obviously, TOX expression is significantly downregulated in multiple cancer types including BLCA (bladder urothelial carcinoma), BRCA (breast invasive carcinoma), COAD (colon adenocarcinoma), ESCA (esophageal carcinoma), KICH (kidney chromophobe), KIRC (kidney renal clear cell carcinoma), KIRP (kidney renal papillary cell carcinoma), LIHC (liver hepatocellular carcinoma), LUAD (lung adenocarcinoma), LUSC (lung squamous cell carcinoma), PRAD (prostate adenocarcinoma), READ (rectum adenocarcinoma), STAD (stomach adenocarcinoma), and THCA (thyroid carcinoma). Only a few of cancer types, CHOL (cholangio carcinoma) and HNSC (head and neck squamous cell carcinoma), upregulated TOX expression in tumor tissues. Due to the lack of adjacent normal tissues, some cancer types are unable to show the change of TOX expression in tumorigenesis. This result reveals that TOX is likely a key tumorigenesis regulator in multiple cancer types and may associate with prognosis.

### 3.2 Prognostic value of TOX in cancers prognosis

To evaluate the prognostic value of TOX in cancers survival, we comprehensively analyzed the correlations between TOX expression and survival in three large cancer databases comprising different extensive samples. The effect of TOX expression to cancers survival was first examined in PrognoScan and the full results are shown in Table S1. Conspicuously, TOX expression is significantly correlated to survival in six cancer types including LUAD, brain glioma, brain astrocytoma, breast cancer, skin melanoma, and bladder transitional cell carcinoma (Table 1). Four datasets (GSE31210, jacob‐00182‐UM, jacob‐00182‐MSK, and GSE13213), comprising 204, 178, 104, and 117 samples, respectively, reveal TOX expression remarkably impacts LUAD overall survival (COX *P* = 7.17e‐05, HR [95% CI] = 0.47 [0.32‐0.68]; COX *P* = .008, HR [95% CI] = 0.55 [0.35‐0.85]; COX *P* = .012, HR [95% CI] = 0.51 [0.30‐0.86]; COX *P* = .017, HR [95% CI] = 0.48 [0.27‐0.88]; COX *P* = .020, HR [95% CI] = 0.78 [0.63‐0.96]) and relapse free survival (COX *P* = 2.07e‐07, HR [95% CI] = 0.45 [0.33‐0.61]; COX *P* = 1.09e‐04, HR [95% CI] = 0.46 [0.31‐0.68]). Similarly, lower TOX expression is also correlated with poor prognosis in brain glioma, brain astrocytoma, breast cancer, and skin melanoma, while only bladder transitional cell carcinoma shows opposite trend. Thus, these initial results suggest that TOX is a potential prognostic factor in survival of brain glioma, brain astrocytoma, breast cancer, skin melanoma, bladder transitional cell carcinoma, and, particularly, LUAD.

**Table 1:** The prognostic potential of TOX in different cancer types by PrognoScan

Dataset	Cancer type	Subtype	Endpoint	Cohort	N	Cox *P*‐value	ln(HR)	HR [95% CI‐low CI‐upp]
GSE31210	Lung cancer	Adenocarcinoma	Relapse free survival	NCCRI	204	2.07E‐07	−0.7965	0.45 [0.33‐0.61]
GSE31210	Lung cancer	Adenocarcinoma	Overall survival	NCCRI	204	7.17E‐05	−0.7541	0.47 [0.32‐0.68]
GSE31210	Lung cancer	Adenocarcinoma	Relapse free survival	NCCRI	204	0.0001	−0.7864	0.46 [0.31‐0.68]
GSE4412‐GPL96	Brain cancer	Glioma	Overall survival	UCLA (1996‐2003)	74	0.0007	−0.6549	0.52 [0.36‐0.76]
jacob‐00182‐UM	Lung cancer	Adenocarcinoma	Overall survival	UM	178	0.0082	−0.6066	0.55 [0.35‐0.85]
GSE31210	Lung cancer	Adenocarcinoma	Overall survival	NCCRI	204	0.0124	−0.6800	0.51 [0.30‐0.86]
jacob‐00182‐MSK	Lung cancer	Adenocarcinoma	Overall survival	MSK	104	0.0174	−0.7242	0.48 [0.27‐0.88]
GSE13213	Lung cancer	Adenocarcinoma	Overall survival	Nagoya (1995‐1999, 2002‐2004)	117	0.0202	−0.2496	0.78 [0.63‐0.96]
GSE4271‐GPL96	Brain cancer	Astrocytoma	Overall survival	MDA	77	0.0219	−0.6093	0.54 [0.32‐0.92]
GSE4412‐GPL96	Brain cancer	Glioma	Overall survival	UCLA (1996‐2003)	74	0.0222	−0.5863	0.56 [0.34‐0.92]
GSE1456‐GPL96	Breast cancer		Disease specific survival	Stockholm (1994‐1996)	159	0.0359	−0.6077	0.54 [0.31‐0.96]
GSE19234	Skin cancer	Melanoma	Overall survival	NYU	38	0.0384	−0.5229	0.59 [0.36‐0.97]
GSE13507	Bladder cancer	Transitional cell carcinoma	Disease specific survival	CNUH	165	0.0387	0.5835	1.79 [1.03‐3.12]
GSE6532‐GPL570	Breast cancer		Relapse free survival	GUYT	87	0.0450	−0.5653	0.57 [0.33‐0.99]
GSE6532‐GPL570	Breast cancer		Distant metastasis free survival	GUYT	87	0.0450	−0.5653	0.57 [0.33‐0.99]
GSE1456‐GPL96	Breast cancer		Overall survival	Stockholm (1994‐1996)	159	0.0458	−0.5206	0.59 [0.36‐0.99]
GSE1379	Breast cancer		Relapse free survival	MGH (1987‐2000)	60	0.0497	−0.7025	0.50 [0.25‐1.00]

Note

This table only shows those of significant difference (cox *P* < .05) and full results are shown in Table S1.

**Figure 2:**
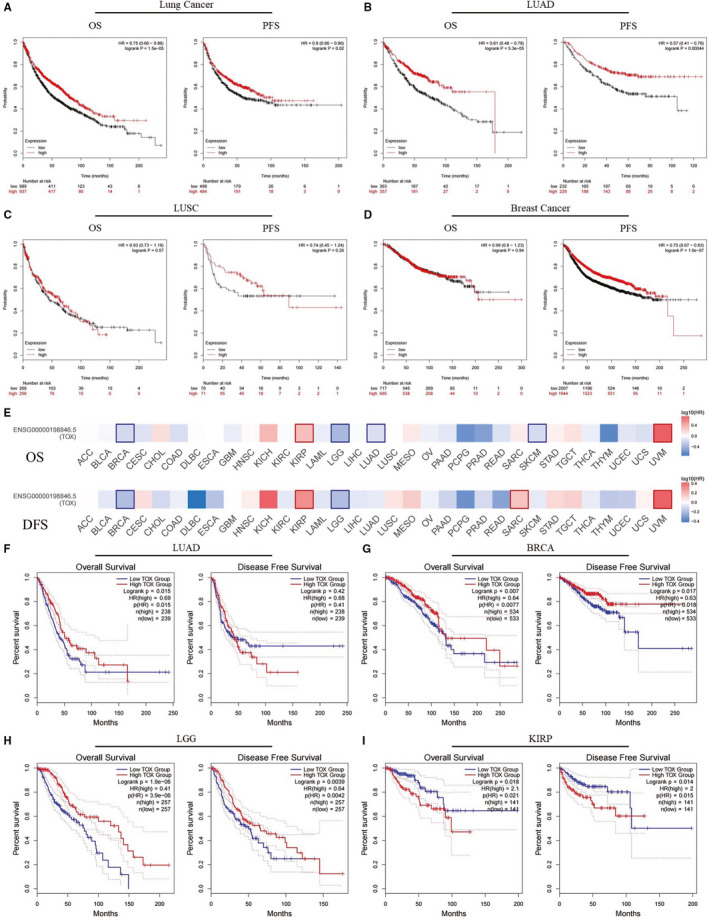
The prognostic potential of TOX in different cancer types evaluated by Kaplan‐Meier Plotter (A‐D) and GEPIA2 (E‐I). OS and PFS survival curves in (A) lung cancer (n = 1926, n = 982), (B) lung adenocarcinoma (n = 720, n = 461), (C) lung squamous cell carcinoma (n = 524, n = 141), and (D) breast cancer (n = 1402, n = 3951). (E) Survival heat map of TOX in 33 TCGA cancer types. The heat map shows the hazard ratios in logarithmic scale (log10) for TOX. The red and blue blocks denote higher and lower risks, respectively. The rectangles with frames mean the significant results in prognostic analysis. OS and DFS survival curves in (F) LUAD (n = 477), (G) BRCA (n = 1067), (H) LGG (n = 514), (I) KIRP (n = 282). OS, overall survival; PFS, progression‐free survival; DFS, disease‐free survival; LUAD, lung adenocarcinoma; LUSC, lung squamous carcinoma; BRCA, breast invasive carcinoma; LGG, brain low grade gliomas; KIRP, kidney renal papillary cell carcinoma

We subsequently analyzed the relationships between TOX expression and prognosis in four large cancer datasets (breast cancer, lung cancer, ovarian cancer, and gastric cancer) provided by Kaplan‐Meier Plotter. Likewise, lower TOX expression correlates with poor prognosis in lung cancer (OS: *P* = 1.5e‐05, HR = 0.75 [0.66‐0.86]; PFS: *P* = .02, HR = 0.8 [0.66‐0.96]), while further subtypes analysis reveals that significant difference only shows in LUAD (OS: *P* = 5.3e‐05, HR = 0.61 [0.48‐0.78]; PFS: *P* = 4.4e‐4, HR = 0.57 [0.41‐0.78]) but not in lung squamous cell carcinoma (OS: *P* = .57, HR = 0.93 [0.73‐1.19]; PFS: *P* = .26, HR = 0.74 [0.45‐1.24]) (Figure 2A‐C). Progress free survival of breast cancer shows similar trend (*P* = 1.9e‐7, HR = 0.75 [0.67‐0.83]) while overall survival shows less difference (*P* = .94, HR = 0.99 [0.8‐1.23]) (Figure 2d). The impact of TOX expression is not detected on OS or PFS of gastric cancer or ovarian cancer (Figure S1A,B).

Base on the results hereinabove, the prognostic potential of TOX was verified using the RNA sequencing expression data of 33 cancer types from TCGA project in GEPIA2. Figure 2e displays the impact of TOX expression on survival in different cancer types. Notably, poor overall survival in LUAD is also correlated with lower TOX expression (*P* = .015, HR = 0.69) (Figure 2f). However, the significant trend does not show in disease‐free survival (*P* = .42, HR = 0.88). With the influence of TOX expression, the significant differences of survival are detected both in BRCA (breast invasive carcinoma) and LGG (brain lower grade glioma) (Figure 2g‐h). Interestingly, the KIRP (kidney renal papillary cell carcinoma) patients with lower TOX expression have better overall as well as disease free survival while the TOX expression of tumor tissues is also lower than normal (Figures 1 and 2I). UVM (uveal melanoma) also shows similar characteristic but the TOX expression change is unknown because of unsuitability to compare the different TOX expression between UVM and normal tissues (Figure 1; Figure S1C). Moreover, TOX expression makes significant differences to the overall survival in SKCM (skin cutaneous melanoma) and the disease‐free survival in SARC (sarcoma) (Figure S1D,E).

These results strongly highlight the prognostic value of TOX in certain types of cancers including breast cancer, brain glioma, and, specifically, LUAD. We further analyzed the prognostic potential of TOX in various subtypes of LUAD (Table 2). Obviously, TOX expression is significantly correlated with survival in early stage or ever‐smoking LUAD. However, the effect of TOX in advanced‐stage LUAD is unknown due to the lack of enough samples. Recently, TMB has been reported as a prognostic factor in specific cancer types after immunotherapy. Thus, we also examined TOX expression in different TMB status of LUAD and found that only in low‐TMB status TOX would be a prognosis‐related biomarker. In summary, these works convincingly revealed the outstanding prognostic potential of TOX in LUAD.

**Table 2:** The prognostic potential of TOX in different subtypes of LUAD by Kaplan‐Meier Plotter

Subtypes			OS		PFS	
			*P*‐value	HR	*P*‐value	HR
Stage		1	**3.10E‐05**	0.43 (0.28‐0.65)	.0534	0.62 (0.38‐1.01)
		2	**.0007**	0.43 (0.26‐0.71)	**.0259**	0.54 (0.31‐0.94)
		3	.6023	1.31 (0.47‐3.68)	—	—
		4	—	—	—	—
AJCC stage	T	1	**.0233**	0.49 (0.27‐0.92)	.2889	0.42 (0.08‐2.18)
		2	**.0084**	0.48 (0.27‐0.84)	.3266	0.73 (0.39‐1.37)
		3	—	—	—	—
		4	—	—	—	—
	N	0	.0555	0.63 (0.39‐1.02)	.8004	0.91 (0.42‐1.96)
		1	**.0003**	0.21 (0.08‐0.52)	.1503	0.51 (0.2‐1.29)
		2	—	—	—	—
	M	0	**.0002**	0.47 (0.31‐0.7)	.1023	0.62 (0.35‐1.11)
		1	—	—	—	—
Gender		Female	**3.00E‐06**	0.4 (0.27‐0.59)	.1744	0.73 (0.46‐1.15)
		Male	**.0003**	0.55 (0.39‐0.76)	**2.20E‐05**	0.38 (0.24‐0.61)
Smoke history		Yes	**2.50E‐05**	0.35 (0.21‐0.58)	**.0012**	0.48 (0.31‐0.76)
		No	.3999	0.71 (0.31‐1.59)	.4853	0.81 (0.44‐1.48)
TMB^a^		High	.2373	0.77 (0.5‐1.19)	.4984	1.23 (0.68‐2.23)
		Low	**.0027**	0.53 (0.35‐0.81)	**.0294**	0.51 (0.27‐0.95)

Note

—, Lack of enough samples and unsuitable to be analyzed. Bold values indicate *P* < .05.

^a^ This subtype was analyzed using lung adenocarcinoma cohort of pan‐cancer database and other subtypes were analyzed using lung cancer database.

### 3.3 TOX correlates with immune infiltration level in LUAD

**Figure 3:**
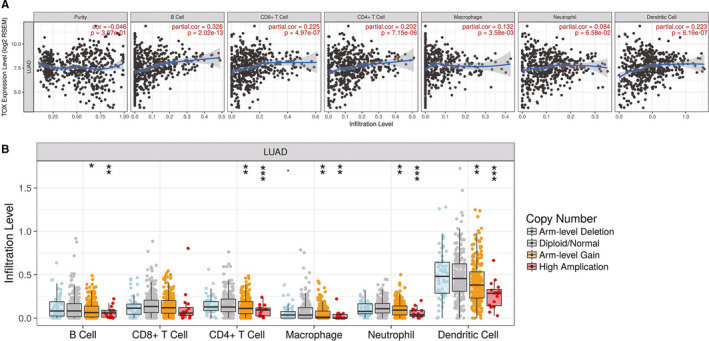
The correlation between TOX and immune infiltration level in LUAD. A, The correlations between TOX expression and the immune infiltrations of tumor purity, B cells, CD8^+^ T cells, CD4^+^ T cells, macrophages, neutrophils, and dendritic cells. B, The comparison of tumor‐infiltration levels in LUAD with different somatic copy number alterations for TOX. SCNAs (somatic copy number alterations) are defined by GISTIC 2.0, including deep deletion (−2), arm‐level deletion (−1), diploid/normal (0), arm‐level gain (1), and high amplification (2). *P*‐value Significant Codes: 0 ≤ *** < .001 ≤ ** < .01 ≤ * < .05 ≤. < .1

Previous studies have indicated the crucial role of TOX in tumor immunity. In order to investigate whether TOX could be an effective biomarker for immunotherapy response prediction or enhancements, we analyzed the relationships between TOX expression and immune infiltration level in LUAD (Figure 3A). Surprisingly, high TOX expression is correlated with high immune infiltration levels of most of the immune cell populations, including B cells (*P* = 2.02e‐13, partial.cor = .326), CD8^+^ T cells (*P* = 4.97e‐07, partial.cor = .225), CD4^+^ T cells (*P* = 7.15e‐06, partial.cor = .202), macrophages (*P* = 3.58e‐03, partial.cor = .132) and dendritic cells (*P* = 6.16e‐07, partial.cor = .223). Only the neutrophil infiltration level shows no significant connection with TOX expression in LUAD (*P* = 6.58e‐02, partial.cor = .084). Moreover, a minority of cancer types, such as BRCA, LGG and SKCM, also were found that their immune infiltration levels are significantly related to TOX (Table S2). We further compared the tumor infiltration level in LUAD with different somatic copy number alterations (SCNA) of TOX (Figure 3B). Notably, normal copy number or deletions spanning the TOX gene locus correlate with increased immune cell infiltration except CD8^+^ T cell. The detailed SCNA analysis of TOX for various cancers is displayed in Table S3. Thus, notwithstanding there is no remarkable correlation between SCNA and immune infiltration in CD8^+^ T cell, our works demonstrated the important impact of TOX on immune infiltration level, particularly for B cells and CD8^+^ cells, in LUAD.

### 3.4 Correlation between TOX and gene markers of immune cells

For the sake of revealing the more specific links between TOX and tumor immune infiltration, correlation analysis between TOX and gene markers of tumor‐infiltration immune cells of LUAD were performed using TIMER and GEPIA2. Referring to previous researches, we selected the gene markers of common immune cell populations and different functional T cells, including B cells, T cells, CD8^+^ T cells, effector T cells, effector memory T cells, central memory T cells, resident memory T cells, exhausted T cells, resting Treg cells, effector Treg cells, T‐helper 1 (Th1), macrophages, neutrophils, dendritic cells, natural killer cells (NK cells), and mast cells. Table 3 shows the correlation analysis results adjusted by tumor purity in LUAD. It is distinct that TOX significantly correlates with the gene markers of B cells, T cells, CD8^+^ T cells, mast cells, and most of the functional T cells, such as effector T cells, effector memory T cells, central memory T cells, exhausted T cells, and effector Treg cells. Interestingly, these results not only corroborated the critical relationships between TOX and B cells, T cells, and functional T cells as previous studies, but also demonstrated a novel close link between TOX and mast cells while rare relevant researches have been reported.

**Table 3:** The correlations between TOX and gene markers of immune cells in LUAD by TIMER

Immune cell	Gene markers	None		Purity	
		Cor	*P*‐value	Cor	*P*‐value
B cell	BLK	.306	***	.334	***
	CD19	.196	***	.212	***
	FCRL2	.134	*	.144	*
	MS4A1	.279	***	.305	***
	KIAA0125	.158	**	.165	**
	TNFRSF17	.170	**	.170	**
	TCL1A	.194	***	.196	***
	SPIB	.303	***	.336	***
	PNOC	.164	**	.174	**
T cell	CD6	.246	***	.272	***
	CD3D	.189	***	.196	***
	CD3E	.263	***	.297	***
	SH2D1A	.258	***	.282	***
	TRAT1	.300	***	.323	***
	CD3G	.241	***	.258	***
CD8^+^ T cell	CD8A	.176	***	.186	***
	CD8B	.141	*	.136	*
Effector T cell	CX3CR1	.293	***	.287	***
	FGFBP2	.212	***	.204	***
	FCGR3A	.009	.831	−.005	.907
Effector memory T cell	PD‐1 (PDCD1)	.097	*	.099	*
	DUSP4	−.117	*	−.113	.012
	GZMK	.279	***	.298	***
	GZMA	.133	*	.129	*
	IFNG	.022	.611	.014	.764
Central memory T cell	CCR7	.306	***	.337	***
	SELL	.226	***	.245	***
	IL7R	.240	***	.248	***
Resident memory T cell	CD69	.285	***	.302	***
	ITGAE	.033	.450	.024	.601
	CXCR6	.198	***	.205	***
	MYADM	−.071	.106	−.077	.088
Exhausted T cell	TIM‐3 (HAVCR2)	.134	*	.131	*
	TIGIT	.189	***	.206	***
	LAG3	.052	.243	.039	.390
	PD‐1 (PDCD1)	.097	*	.099	*
	CXCL13	.185	***	.194	***
	LAYN	.026	.554	.006	.898
Resting Treg T cell	FOXP3	.146	***	.144	***
	IL2RA	.080	.070	.066	.141
Effector Treg T cell	FOXP3	.146	***	.144	***
	CTLA4	.141	*	.136	*
	CCR8	.201	***	.199	***
	TNFRSF9	.074	.094	.056	.216
Th1	TBX21	.196	***	.207	***
	IFNG	.022	.611	.014	.764
	TNF	.070	.114	.055	.223
Macrophage	CD68	.046	.302	.042	.348
	CD84	.228	***	.233	***
	CD163	.058	.192	.049	.281
	MS4A4A	.142	*	.139	*
Neutrophils	FPR1	.081	.066	.066	.145
	SIGLEC5	.130	*	.123	*
	CSF3R	.139	*	.133	*
	FCAR	−.006	.885	−.003	.952
	FCGR3B	−.039	.380	−.053	.239
	CEACAM3	.006	.895	−.016	.729
	S100A12	−.189	***	−.208	***
Dendritic cell	CCL13	.130	*	.120	*
	CD209	.069	.115	.062	.168
	HSD11B1	.098	.027	.096	.033
Natural killer cell	XCL1	.083	.060	.070	.118
	XCL2	.114	*	.111	.013
	NCR1	.047	.291	.040	.375
Mast cell	TPSB2	.262	***	.269	***
	TPSAB1	.278	***	.281	***
	CPA3	.321	***	.318	***
	MS4A2	.338	***	.341	***
	HDC	.268	***	.271	***

Note

Cor, ρ value of Spearman's correlation. None, correlation without adjustment. Purity, correlation adjusted by tumor purity.

*P*‐value significant codes: 0 ≤ *** < .001 ≤ ** < .01 ≤ * < .01.

To validated above findings, we further analyzed the correlations between TOX expression and gene markers set of immune cells in LUAD as well as normal samples using GEPIA2 (Table 4). Similarly, TOX shows positive relationships to B cells (Cor = .21, *P* < .0001), T cells (Cor = .27, *P* < .0001), mast cells (Cor = .32, *P* < .0001) and multiple functional T cells, especially effector T cells (Cor = .26, *P* < .0001) and central memory T cells (Cor = .32, *P* < .0001). Here, we noticed that TOX has significant interactions with multiple key genes of exhausted T cell comprising PD‐1 (Cor = .11, *P* = .014), TIM‐3 (Cor = .18, *P* < .0001), TIGHT (Cor = .2, *P* < .0001) and CXCL13 (Cor = .16, *P* < .001), which play critical role on current cancer immunotherapy. Notwithstanding other immune cells including macrophages, neutrophils, dendritic cells, and NK cells also show significant difference in the analysis, their strengths of the correlations are weaker than aforementioned immune cells. As contrasts, the correlations between TOX and gene markers set of immune cells all show no significant difference in normal samples.

**Table 4:** The correlations between TOX and gene markers of immune cells in LUAD and normal by GEPIA2

Immune cell	Gene markers	Tumor		Tumor‐Sum		Normal		Normal‐Sum	
		Cor	*P*‐value	Cor	*P*‐value	Cor	*P*‐value	Cor	*P*‐value
B cell	BLK	.3	***	.21	***	.025	.85	−.091	.49
	CD19	.17	**			−.084	.53		
	FCRL2	.092	.044			.0078	.95		
	MS4A1	.27	***			.0016	.99		
	KIAA0125	.079	.085			.0011	.99		
	TNFRSF17	.1	.025			−.081	.54		
	TCL1A	.2	***			−.054	.69		
	SPIB	.31	***			−.085	.52		
	PNOC	.13	*			−.063	.64		
T cell	CD6	.26	***	.27	***	.09	.5	.037	.78
	CD3D	.15	**			−.17	.21		
	CD3E	.28	***			.17	.21		
	SH2D1A	.27	***			.047	.73		
	TRAT1	.32	***			−.016	.9		
	CD3G	.27	***			.11	.39		
CD8^+^ T cell	CD8A	.18	***	.17	**	.21	.11	.16	.22
	CD8B	.14	*			.11	.39		
Effector T cell	CX3CR1	.34	***	.26	***	−.11	.39	−.11	.42
	FGFBP2	.22	***			−.041	.76		
	FCGR3A	.082	.071			−.063	.63		
Effector memory T cell	PD‐1 (PDCD1)	.11	.014	.11	.015	.28	.034	−.047	.73
	DUSP4	−.098	.03			.053	.69		
	GZMK	.3	***			.042	.75		
	GZMA	.12	*			−.18	.18		
	IFNG	.037	.41			−.3	.021		
Central memory T cell	CCR7	.33	***	.32	***	.032	.81	.17	.19
	SELL	.26	***			.21	.11		
	IL7R	.29	***			.1	.45		
Resident memory T cell	CD69	.3	***	.24	***	−.081	.54	.028	.84
	ITGAE	.011	.8			−.3	.019		
	CXCR6	.21	***			−.073	.58		
	MYADM	.045	.32			.23	.081		
Exhausted T cell	TIM‐3 (HAVCR2)	.18	***	.17	**	−.042	.75	−.032	.81
	TIGIT	.2	***			.2	.12		
	LAG3	.061	.18			.011	.93		
	PD‐1 (PDCD1)	.11	.014			.28	.034		
	CXCL13	.16	**			−.18	.18		
	LAYN	.084	.065			−.11	.39		
Resting Treg T cell	FOXP3	.18	***	.17	**	.067	.61	.23	.082
	IL2RA	.13	*			.32	.013		
Effector Treg T cell	FOXP3	.18	***	.18	***	.067	.61	.17	.21
	CTLA4	.16	**			.16	.24		
	CCR8	.25	***			.019	.89		
	TNFRSF9	.096	.035			.21	.11		
Th1	TBX21	.23	***	.12	*	.15	.26	−.11	.4
	IFNG	.037	.41			−.3	.021		
	TNF	.094	.04			−.17	.19		
Macrophage	CD68	.13	*	.18	***	.15	.27	.11	.4
	CD84	.3	***			.14	.28		
	CD163	.074	.1			.12	.37		
	MS4A4A	.19	***			−.1	.45		
Neutrophils	FPR1	.13	*	.13	*	.22	.087	.19	.16
	SIGLEC5	.16	**			.1	.43		
	CSF3R	.18	***			.35	*		
	FCAR	.058	.2			.15	.26		
	FCGR3B	.037	.42			.13	.34		
	CEACAM3	.059	.19			.13	.32		
	S100A12	−.18	***			.051	.7		
Dendritic cell	CCL13	.14	*	.18	***	−.079	.55	.032	.81
	CD209	.15	*			.23	.079		
	HSD11B1	.14	*			−0.1	.45		
Natural killer cell	XCL1	.089	.05	.096	.035	−0.2	.14	−.23	.086
	XCL2	.094	.04			−.28	.034		
	NCR1	.086	.058			.15	.27		
Mast cell	TPSB2	.25	***	.32	***	.093	.48	.13	.32
	TPSAB1	.29	***			.13	.31		
	CPA3	.35	***			.099	.46		
	MS4A2	.38	***			.12	.36		
	HDC	.3	***			.21	.12		

Note

Cor, ρ value of Spearman's correlation. Tumor, single gene marker correlation analysis in LUAD tissue. Tumor‐Sum, gene markers set correlation analysis in LUAD tissue. Normal, single gene marker correlation analysis in normal tissue. Normal‐Sum, gene markers set correlation analysis in normal tissue.

*P*‐value significant codes: 0 ≤ *** < .001 ≤ ** < .01 ≤ * < .01.

These results further confirmed that TOX positively participates in multiple pivotal immune cells infiltration, especially B cells and T cells, of LUAD as a critical factor. What stands out is the remarkable interactions between TOX and multiple key gene of exhausted T cells, suggesting the considerable potential value of TOX applied on immunotherapy for LUAD. Additionally, we found a novel connection between TOX and mast cells in LUAD which also shows its potential value and it is worth further investigation of underlying mechanism.

### 3.5 TOX highly expresses in exhausted T cells in LUAD

**Figure 4:**
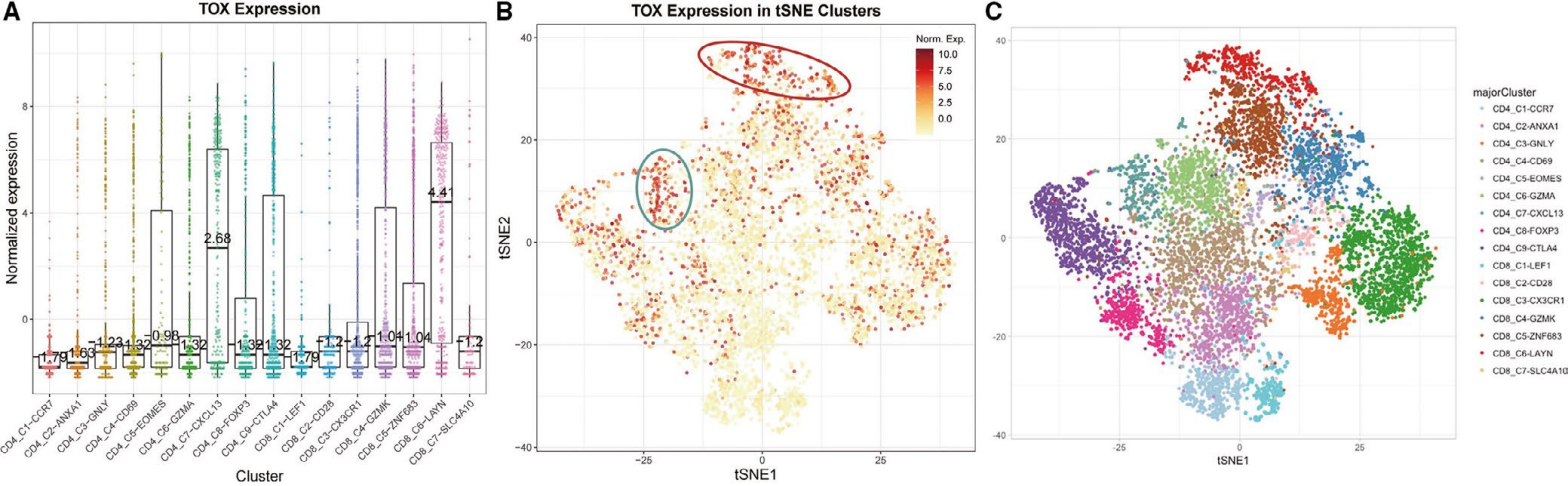
The expression of TOX in T cells of LUAD by single‐cell sequencing. A, The normalized expression of TOX in different T cells populations. B, The expression of TOX in individual T cells. Every dot represents one cell and the color of dot represents the expression levels. C, The distribution of major T cells cluster in t‐SNE plot

Our findings have demonstrated that TOX plays an important role on T cells and functional T cells infiltration in LUAD. In order to investigate the specific interaction between TOX, T cells, and functional T cells, we acquired the single‐cell RNA‐seq data for T cells in LUAD in an online open database based on a previous study.^29^ By applying unsupervised clustering based on t‐SNE+densityClust, the database identified 16 main clusters. The expression of TOX in different populations after normalized was showed in Figure 4A. Interestingly, TOX is of significant high expression in CD4‐C7‐CXCL13 and CD8‐C6‐LAYN populations compared to others, which representing exhausted CD4^+^ T cells and exhausted CD8^+^ T cells, respectively. The t‐SNE plot shows two T cells enrichment regions with high TOX expression, highly overlapping with CD4‐C7‐CXCL13 and CD8‐C6‐LAYN clusters (Figure 4B,C). Previous articles have reported the underlying relationship between TOX and exhausted T cells and our above findings future verified their close link by single‐cell sequencing analysis.

## 4 DISCUSSION

TOX is a nuclear DNA‐binding factor and an HMG (high mobility box) protein playing an important role on development of various aspects of lymphocytes especially T cells.^18^, ^19^, ^20^ Recently, several studies have demonstrated the crucial role of TOX in tumor‐specific T cells differentiation and CD8^+^ T cell exhaustion^21^, ^22^, ^34^ and specific regulation mechanism in some diseases, such as T‐ALL (T‐cell acute lymphoblastic leukemia) and encephalitogenic.^35^, ^36^ However, the relationships among TOX expression, prognosis, immune infiltration, and T cells function in different cancers types have not been comprehensively investigated. Here, we acquired and analyzed extensive tumor samples from several large databases and found that TOX expression level is correlated with prognosis in multiple types of tumor, particularly LUAD. Future research revealed the positively connection between TOX expression and immune infiltration level in LUAD. By analyzing genes expression correlations and single‐cell RNA‐seq for T cells, we confirmed TOX has significant interaction with most of the functional T cells especially exhausted T cells in LUAD. Thus, we demonstrated TOX is a potential prognosis‐related biomarker in LUAD and provided a novel direction to understand the correlations between TOX, immune infiltration and T cells function.

In this work, we investigated the expression level of TOX and its prognostic potential in various types of tumor using several databases comprised a large number of tumor and normal samples. Most of the cancer types, such as BRCA, KIRP, LUAD, and LUSC, are detected significantly downregulated TOX expression compared to normal tissue or metastasis and only two types of cancer (CHOL and HNSC) show opposite trend, indicating a strong possibility that TOX is an important survival prognostic factor. Subsequently, the comprehensive prognostic landscape of TOX in different types of tumor proved our hypothesis. After analyzing extensive variety of tumor patients’ survival data from several large databases, we confirmed low expression level of TOX is significantly correlated with poor prognosis in multiple cancers types comprising breast cancer, brain low grade glioma, and particularly LUAD (Figure 2A‐I). The underlying mechanism may be the indispensable role of TOX in the development of innate lymphoid cells and regulation of tumor‐specific T cells, which exerts an important effect on tumor microenvironment.^21^, ^37^, ^38^, ^39^ Interestingly, KIRP with low expression level of TOX has better prognosis while the TOX expression of tumor tissues is also lower than normal. This unusual trend may imply certain specific unknown mechanism and require further investigation. Because of the excellent prognostic potential of TOX in LUAD, after examining the correlations between TOX expression and various subtypes of LUAD, we found that the survival of early stage or ever‐smoking LUAD is significant correlated with TOX expression while those of advanced‐stage remain unknown due to lack of enough samples (Table 2). Because TMB was extensively investigated and has been confirmed that it correlates with immunotherapy response and prognosis in many cancer types,^17^, ^40^ in order to investigate whether different TMB status could affect the prognostic power of TOX, we examined expression level of TOX in different TMB status of LUAD and found that only in low‐TMB status TOX could show statistical difference. Therefore, a model associated with TOX and TMB may have more powerful prediction capabilities, which is also worth further research. In brief, these findings convincingly suggest that TOX a prognosis‐related biomarker in LUAD.

In consideration of the critical role of TOX in immune system and its outstanding prognostic value for LUAD, we analyzed the relationships between TOX and immune infiltration level in LUAD (Figure 3A). The results showed that high expression level of TOX is significantly correlated with high immune infiltration levels of most of the immune cell populations including CD4^+^ T cells, macrophages, dendritic cells and especially, B cells and CD8^+^ T cells, which have stronger correlation level. Although different SCNA of TOX showed no significant impact in the immune infiltration level of CD8^+^ T cells in LUAD (Figure 3B), the close links among TOX, B cells and CD8^+^ T cells aroused our interest. Further investigations for correlations between TOX and gene markers of immune cells revealed that TOX has compelling interactions with not only most of the immune cells but also various functional T cells, such as exhausted T cells, effector T cells, and central memory T cells (Tables 3 and 4). Due to the exhaustion of T cells is major cause of inefficient antitumor immunity,^41^, ^42^, ^43^ the strategies to prevent the development of exhausted T cells are the core of cancer immunotherapy. We noticed that high expression level of TOX is positively correlated with multiple key genes of exhausted T cells comprised PD‐1, TIM‐3, TIGHT, and CXCL13, which are current therapeutic targets or have crucial role on immunotherapy.^44^, ^45^ After analyzing single‐cell RNA‐seq data for T cells in LUAD, we observed that TOX is highly enriched in exhausted CD4^+^ and CD8^+^ T cells populations, which verified the vital role of TOX on regulating the development of T cells exhaustion as reported in previous article.^21^

Interestingly, our findings suggest that TOX has dual functions that high expression level of TOX is positively correlated with better prognosis in multiple cancer types included LUAD and, meanwhile, induces T cells exhaustion which causes the inefficiency of antitumor immunity. In fact, these two especial and seems opposite trends are not contradictory and recent studies provided some insights may explain the underlying mechanisms. On the one hand, TOX is required for the differentiation of common lymphoid progenitors into innate lymphoid cell lineage‐restricted cells as a transcriptional regulator.^18^, ^20^, ^37^ TOX deficiency leads to early defects in progenitor cell survival or proliferation, as well as innate lymphoid cell differentiation at later stage. The expression of TOX is an indispensable factor for the development and maintenance of T cells. On the other hand, TOX is a vital inducer of canonical features of T cells exhaustion and an initiator of the exhausted T cells specific epigenetic program.^22^, ^34^ Following chronic antigenic stimulation, TOX enforces the transcriptional profile of dysfunctional T cells and promotes the acquisition of T cells exhausted phenotype. Therefore, TOX plays critical but different role on the development of normal immunity and the regulation of tumor microenvironment, which is needed for identification in specific stage.

An additional finding in our works is the novel relationship between TOX and mast cells in LUAD, which is rarely reported in previous studies. Mast cells not only have effector functions in TH2‐skewed allergic and autoimmune inflammation but also can promote adequate inflammatory responses and cooperate with dendritic cells in T‐cell activation.^46^ Recently, several researches demonstrated the nonnegligible impact of mast cells in the conformation of tumor microenvironment and the promotion for specific cancer.^47^, ^48^ We found that high expression level of TOX positively correlates with the expression of pivotal gene markers (TPSB2, TPSAB1, CPA3, MS4A2, and HDC) of mast cells, indicating its potential value and it is worth further study of the underlying mechanism.

The limitations of our study were as follows: First, our results could not be validated due to the absence of experiment. For the sake of validating and further understanding TOX function for immune oncology, we plan to compare the effect of checkpoint blockade between TOX knockdown model and normal both in vivo and in vitro. Second, our study shows that TOX probably correlates to immunotherapy response in LUAD but currently we have no suitable population data to prove this guess. We plan to collect relevant data of patients receiving immunotherapy in our institution and hope to get enough sample size to perform survival analysis. Finally, the data used in our study were accessed from public databases while the quality of the data could not be well appraised.

In summary, our findings suggest that TOX is a prognosis‐related biomarker for multiple cancer types particularly LUAD. Increased TOX expression correlates with high immune infiltration levels in B cells, CD8^+^ T cells, CD4^+^ T cells, and most of the functional T cells. Notwithstanding playing crucial role on the development of immunity, TOX also highly correlates with exhausted T cells and is probable a critical regulator promoted T cells exhaustion in LUAD. Detecting TOX expression may help to predict prognosis and regulating TOX expression in exhausted T cells may provide a new strategy in maximizing immunotherapy efficacy for LUAD patients.

## CONFLICT OF INTEREST

The authors declare no potential conflicts of interest.

## AUTHOR CONTRIBUTIONS

SD, CR, and LG contributed conception and design of the study. LG, XL, RL, and YC participated in data analysis. LG performed data curation and visualization. LG was major contributor in writing the manuscript. All authors contributed to manuscript revision, read, and approved the final version.

## Supporting information

Fig S1

Table S1‐S3

## Data Availability

The data that support the findings of this study are available in TIMER (https://cistrome.shinyapps.io/timer/), PrognoScan (http://dna00.bio.kyutech.ac.jp/PrognoScan/), Kaplan‐Meier Plotter (http://kmplot.com/), GEPIA2 (http://gepia2.cancer‐pku.cn/), and http://lung.cancer‐pku.cn/.
