# Supplementary material for: TOX correlates with prognosis, immune infiltration, and T cells exhaustion in lung adenocarcinoma
Source: Cancer Med. 2020 Jul 23;9(18):6694–709. doi: 10.1002/cam4.3324 (PMC7520261; doi:10.1002/cam4.3324)
Supplement: Supplementary file 1 — Fig S1 [file CAM4-9-6694-s001.pdf]

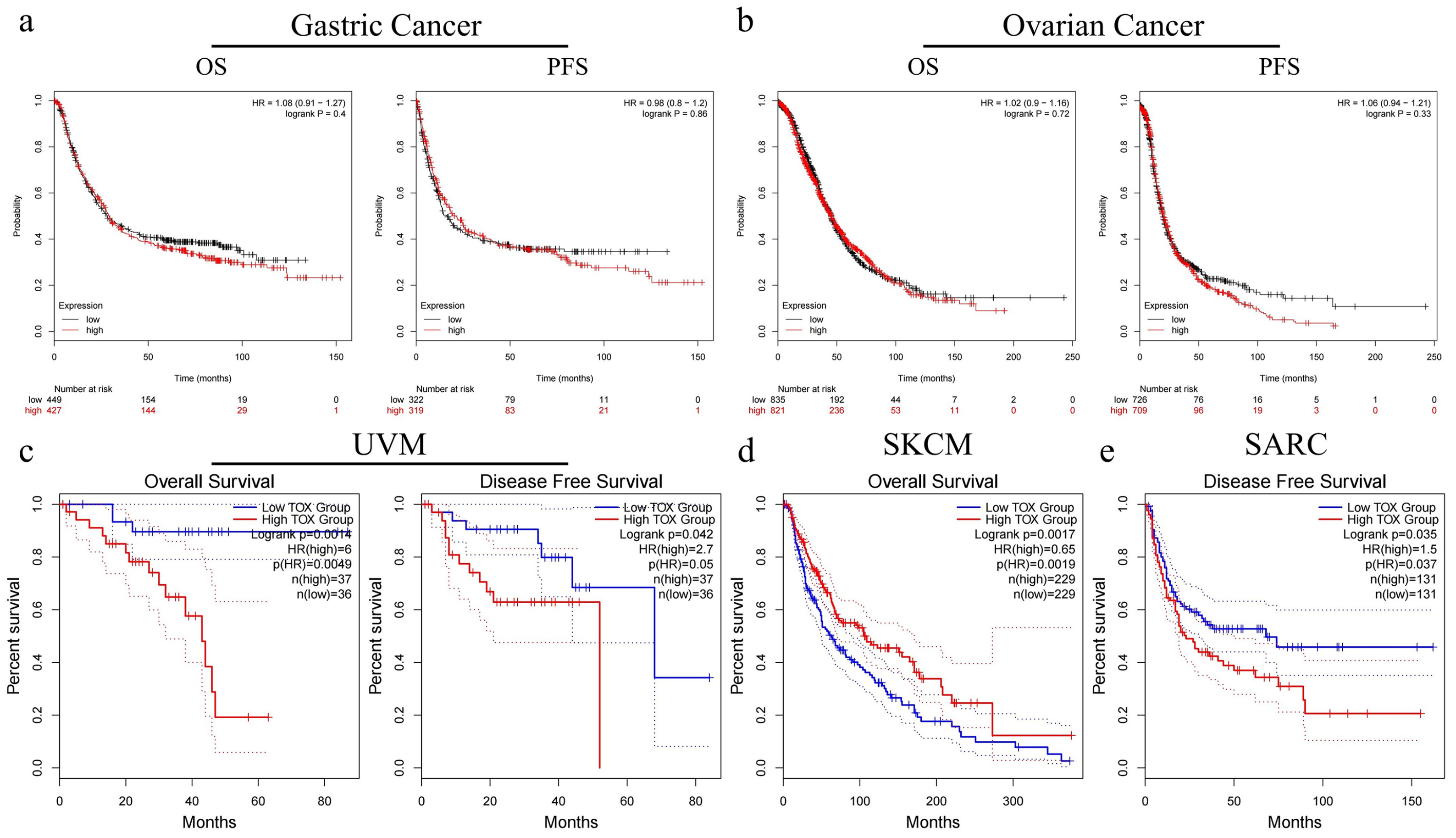

a
Gastric Cancer
OS
PFS
HR = 1.08 (0.91 − 1.27)
logrank P = 0.4
HR = 0.98 (0.8 − 1.2)
logrank P = 0.86
Probability
Time (months)
Expression
low
high
Number at risk
low 449 154 19 0
high 427 144 29 1
low 322 79 11 0
high 319 83 21 1
b
Ovarian Cancer
OS
PFS
HR = 1.02 (0.9 − 1.16)
logrank P = 0.72
HR = 1.06 (0.94 − 1.21)
logrank P = 0.33
low 835 192 44 7 2 0
high 821 236 53 11 0 0
low 726 76 16 5 1 0
high 709 96 19 3 0 0
c
UVM
Overall Survival
Low TOX Group
High TOX Group
Logrank p=0.0014
HR(high)=6
p(HR)=0.0049
n(high)=37
n(low)=36
Disease Free Survival
Logrank p=0.042
HR(high)=2.7
p(HR)=0.05
n(high)=37
n(low)=36
Percent survival
Months
d
SKCM
Overall Survival
Logrank p=0.0017
HR(high)=0.65
p(HR)=0.0019
n(high)=229
n(low)=229
e
SARC
Disease Free Survival
Logrank p=0.035
HR(high)=1.5
p(HR)=0.037
n(high)=131
n(low)=131
