# Supplementary material for: TOX correlates with prognosis, immune infiltration, and T cells exhaustion in lung adenocarcinoma
Source: Cancer Med. 2020 Jul 23;9(18):6694–709. doi: 10.1002/cam4.3324 (PMC7520261; doi:10.1002/cam4.3324)
Supplement: Supplementary file 2 — Table S1‐S3 [file CAM4-9-6694-s002.pdf]

Supplementary Table 1. The effect of TOX expression to cancers survival by PrognoScan.

DATASET	CANCER TYPE	SUBTYPE	ENDPOINT	COHORT	N	CORRECTED P-VALUE	ln(HR-high / HR-low)	COX P-VALUE	ln(HR)	HR [95% CI-low CI-upp]
GSE5287	Bladder cancer		Overall Survival	Aarhus (1995-2004)	30	-	-0.914659	0.407075	-1.05085	0.35 [0.03 - 4.19]
GSE5287	Bladder cancer		Overall Survival	Aarhus (1995-2004)	30	-	-0.647532	0.894285	0.0555922	1.06 [0.47 - 2.40]
GSE13507	Bladder cancer		Overall Survival	CNUH	165	0.124826	0.643802	0.50948	0.156086	1.17 [0.74 - 1.86]
GSE13507	Bladder cancer	Transitional cell carcinoma	Disease Specific Survival	CNUH	165	0.0119049	1.20109	0.0387098	0.583462	1.79 [1.03 - 3.12]
GSE12417-GPL96	Blood cancer	AML	Overall Survival	AMLCG (1999-2003)	163	0.15452	0.526661	0.197834	0.129466	1.14 [0.93 - 1.39]
GSE12417-GPL96	Blood cancer	AML	Overall Survival	AMLCG (1999-2003)	163	-	0.276676	0.572628	0.200567	1.22 [0.61 - 2.45]
GSE12417-GPL570	Blood cancer	AML	Overall Survival	AMLCG (2004)	79	-	-0.482153	0.65719	0.0568367	1.06 [0.82 - 1.36]
GSE12417-GPL570	Blood cancer	AML	Overall Survival	AMLCG (2004)	79	0.331329	1.10075	0.31986	0.259224	1.30 [0.78 - 2.16]
GSE5122	Blood cancer	AML	Overall Survival	San Diego	58	-	-0.522784	0.914931	-0.0268941	0.97 [0.59 - 1.59]
GSE5122	Blood cancer	AML	Overall Survival	San Diego	58	-	-0.453435	0.51035	-0.0926751	0.91 [0.69 - 1.20]
GSE8970	Blood cancer	AML	Overall Survival	San Diego	34	-	0.918409	0.248769	0.363023	1.44 [0.78 - 2.66]
GSE8970	Blood cancer	AML	Overall Survival	San Diego	34	-	0.750904	0.848625	0.0618267	1.06 [0.56 - 2.01]
GSE4475	Blood cancer	B-cell lymphoma	Overall Survival	Berlin (2003-2005)	158	-	0.626571	0.538256	-0.167397	0.85 [0.50 - 1.44]
GSE4475	Blood cancer	B-cell lymphoma	Overall Survival	Berlin (2003-2005)	158	0.0873564	-0.73368	0.085638	-0.157159	0.85 [0.71 - 1.02]
E-TABM-346	Blood cancer	DLBCL	Event Free Survival	GELA (1998-2000)	53	-	-0.647806	0.433173	-0.141106	0.87 [0.61 - 1.24]
E-TABM-346	Blood cancer	DLBCL	Overall Survival	GELA (1998-2000)	53	-	-0.739008	0.321044	-0.244983	0.78 [0.48 - 1.27]
E-TABM-346	Blood cancer	DLBCL	Overall Survival	GELA (1998-2000)	53	0.351495	-0.804017	0.454703	-0.141399	0.87 [0.60 - 1.26]
E-TABM-346	Blood cancer	DLBCL	Event Free Survival	GELA (1998-2000)	53	0.345292	-1.01893	0.127182	-0.355502	0.70 [0.44 - 1.11]
GSE16131-GPL96	Blood cancer	Follicular lymphoma	Overall Survival	NCI (1974-2001)	180	-	-0.365077	0.956611	-0.0087616	0.99 [0.72 - 1.36]
GSE16131-GPL96	Blood cancer	Follicular lymphoma	Overall Survival	NCI (1974-2001)	180	0.491578	-0.502126	0.3094	-0.121864	0.89 [0.70 - 1.12]
GSE2658	Blood cancer	Multiple myeloma	Disease Specific Survival	Arkansas	559	0.409958	-0.850534	0.867692	-0.0138479	0.99 [0.84 - 1.16]
GSE2658	Blood cancer	Multiple myeloma	Disease Specific Survival	Arkansas	559	0.368575	-0.772225	0.372878	-0.0808553	0.92 [0.77 - 1.10]
GSE4271-GPL96	Brain cancer	Astrocytoma	Overall Survival	MDA	77	-	-0.50651	0.620495	-0.0803536	0.92 [0.67 - 1.27]
GSE4271-GPL96	Brain cancer	Astrocytoma	Overall Survival	MDA	77	0.0840459	-0.778709	0.0219356	-0.609338	0.54 [0.32 - 0.92]
GSE7696	Brain cancer	Glioblastoma	Overall Survival	Lausanne	70	-	-0.513583	0.301639	-0.14431	0.87 [0.66 - 1.14]
GSE7696	Brain cancer	Glioblastoma	Overall Survival	Lausanne	70	0.299848	-0.604308	0.354544	-0.257596	0.77 [0.45 - 1.33]
MGH-glioma	Brain cancer	Glioma	Overall Survival	CBTTB, MGH, BWH, CH	50	0.356888	-0.832587	0.158716	-0.763598	0.47 [0.16 - 1.35]
GSE4412-GPL96	Brain cancer	Glioma	Overall Survival	UCLA (1996-2003)	74	0.0507647	-0.950625	0.022201	-0.586317	0.56 [0.34 - 0.92]
GSE4412-GPL96	Brain cancer	Glioma	Overall Survival	UCLA (1996-2003)	74	0.00214727	-2.06988	0.000729339	-0.654899	0.52 [0.36 - 0.76]
GSE16581	Brain cancer	Meningioma	Overall Survival	UCLA	67	-	-15.3567	0.366924	-1.12212	0.33 [0.03 - 3.73]
GSE16581	Brain cancer	Meningioma	Overall Survival	UCLA	67	-	-1.28826	0.333829	-0.321989	0.72 [0.38 - 1.39]
GSE19615	Breast cancer		Distant Metastasis Free Survival	DF/HCC	115	0.465481	-15.4059	0.520231	-0.368669	0.69 [0.22 - 2.13]
GSE19615	Breast cancer		Distant Metastasis Free Survival	DF/HCC	115	-	0.997147	0.197299	0.579299	1.78 [0.74 - 4.31]
GSE3143	Breast cancer		Overall Survival	Duke	158	-	0.59123	0.959749	0.00891945	1.01 [0.71 - 1.43]
GSE7849	Breast cancer		Disease Free Survival	Duke (1990-2001)	76	-	1.06352	0.545998	0.170968	1.19 [0.68 - 2.07]
GSE12276	Breast cancer		Relapse Free Survival	EMC	204	0.149094	0.382526	0.104956	0.132487	1.14 [0.97 - 1.34]
GSE12276	Breast cancer		Relapse Free Survival	EMC	204	0.482477	-0.283119	0.42096	-0.0576767	0.94 [0.82 - 1.09]
GSE6532-GPL570	Breast cancer		Relapse Free Survival	GUYT	87	0.203113	-15.3262	0.0450446	-0.565315	0.57 [0.33 - 0.99]
GSE6532-GPL570	Breast cancer		Relapse Free Survival	GUYT	87	0.137935	-1.01747	0.108683	-1.87618	0.15 [0.02 - 1.52]
GSE6532-GPL570	Breast cancer		Distant Metastasis Free Survival	GUYT	87	0.203113	-15.3262	0.0450446	-0.565315	0.57 [0.33 - 0.99]
GSE6532-GPL570	Breast cancer		Distant Metastasis Free Survival	GUYT	87	0.137935	-1.01747	0.108683	-1.87618	0.15 [0.02 - 1.52]
GSE9195	Breast cancer		Distant Metastasis Free Survival	GUYT2	77	0.212113	-1.54399	0.68265	-0.699927	0.50 [0.02 - 14.23]
GSE9195	Breast cancer		Distant Metastasis Free Survival	GUYT2	77	0.348256	-1.29591	0.477198	0.232265	1.26 [0.66 - 2.39]
GSE9195	Breast cancer		Relapse Free Survival	GUYT2	77	0.384376	-1.13038	0.757644	0.0953834	1.10 [0.60 - 2.02]
GSE9195	Breast cancer		Relapse Free Survival	GUYT2	77	-	-0.813165	0.758058	0.433275	1.54 [0.10 - 24.29]
GSE12093	Breast cancer		Distant Metastasis Free Survival	IO, NCI, TUM, CCF (1992-2000)	136	-	0.824707	0.482179	-0.192839	0.82 [0.48 - 1.41]
GSE12093	Breast cancer		Distant Metastasis Free Survival	IO, NCI, TUM, CCF (1992-2000)	136	-	-0.745513	0.582749	-0.125262	0.88 [0.56 - 1.38]
GSE11121	Breast cancer		Distant Metastasis Free Survival	Mainz (1988-1998)	200	0.377462	0.703638	0.915507	0.0261577	1.03 [0.63 - 1.66]
GSE11121	Breast cancer		Distant Metastasis Free Survival	Mainz (1988-1998)	200	-	-0.655424	0.73455	-0.0890043	0.91 [0.55 - 1.53]
GSE1378	Breast cancer		Relapse Free Survival	MGH (1987-2000)	60	-	0.512303	0.851599	-0.0610503	0.94 [0.50 - 1.78]
GSE1379	Breast cancer		Relapse Free Survival	MGH (1987-2000)	60	9.73E-07	-2.28945	0.0496781	-0.702473	0.50 [0.25 - 1.00]
GSE2034	Breast cancer		Distant Metastasis Free Survival	Rotterdam (1980-1995)	286	-	0.383196	0.936178	-0.0121586	0.99 [0.73 - 1.33]
GSE2034	Breast cancer		Distant Metastasis Free Survival	Rotterdam (1980-1995)	286	-	-0.293596	0.852713	-0.0331336	0.97 [0.68 - 1.37]
GSE1456-GPL96	Breast cancer		Relapse Free Survival	Stockholm (1994-1996)	159	-	0.568427	0.835927	0.0496413	1.05 [0.66 - 1.68]
GSE1456-GPL96	Breast cancer		Overall Survival	Stockholm (1994-1996)	159	0.00507125	-1.15866	0.0457792	-0.520595	0.59 [0.36 - 0.99]
GSE1456-GPL96	Breast cancer		Disease Specific Survival	Stockholm (1994-1996)	159	-	-0.853497	0.529168	-0.154325	0.86 [0.53 - 1.39]
GSE1456-GPL96	Breast cancer		Overall Survival	Stockholm (1994-1996)	159	-	-0.599145	0.672811	-0.0921011	0.91 [0.59 - 1.40]
GSE1456-GPL96	Breast cancer		Relapse Free Survival	Stockholm (1994-1996)	159	0.079857	-0.885977	0.40485	-0.248723	0.78 [0.43 - 1.40]
GSE1456-GPL96	Breast cancer		Disease Specific Survival	Stockholm (1994-1996)	159	0.00391594	-1.39472	0.0359103	-0.607672	0.54 [0.31 - 0.96]

GSE7378	Breast cancer		Disease Free Survival	UCSF	54	-	-1.16732	0.267177	-2.84946	0.06 [0.00 - 8.88]
GSE7378	Breast cancer		Disease Free Survival	UCSF	54	0.299063	-15.8312	0.0936868	-2.8669	0.06 [0.00 - 1.62]
E-TABM-158	Breast cancer		Overall Survival	UCSF, CPMC (1989-1997)	117	0.397645	-1.40607	0.240218	-0.295838	0.74 [0.45 - 1.22]
E-TABM-158	Breast cancer		Distant Metastasis Free Survival	UCSF, CPMC (1989-1997)	117	-	-0.685174	0.524946	-0.185641	0.83 [0.47 - 1.47]
E-TABM-158	Breast cancer		Relapse Free Survival	UCSF, CPMC (1989-1997)	117	0.205722	1.60813	0.724756	0.338289	1.40 [0.21 - 9.22]
E-TABM-158	Breast cancer		Disease Specific Survival	UCSF, CPMC (1989-1997)	117	0.334137	1.93119	0.89824	0.148104	1.16 [0.12 - 11.22]
E-TABM-158	Breast cancer		Distant Metastasis Free Survival	UCSF, CPMC (1989-1997)	117	-	0.752642	0.470433	0.829462	2.29 [0.24 - 21.80]
E-TABM-158	Breast cancer		Overall Survival	UCSF, CPMC (1989-1997)	117	0.205722	1.60813	0.724756	0.338289	1.40 [0.21 - 9.22]
E-TABM-158	Breast cancer		Disease Specific Survival	UCSF, CPMC (1989-1997)	117	0.153177	-0.968461	0.102188	-0.631566	0.53 [0.25 - 1.13]
E-TABM-158	Breast cancer		Relapse Free Survival	UCSF, CPMC (1989-1997)	117	0.397645	-1.40607	0.240218	-0.295838	0.74 [0.45 - 1.22]
GSE3494-GPL96	Breast cancer		Disease Specific Survival	Uppsala (1987-1989)	236	0.488775	-0.52647	0.995754	-0.00145953	1.00 [0.58 - 1.71]
GSE3494-GPL96	Breast cancer		Disease Specific Survival	Uppsala (1987-1989)	236	-	0.394543	0.486322	0.180981	1.20 [0.72 - 1.99]
GSE4922-GPL96	Breast cancer		Disease Free Survival	Uppsala (1987-1989)	249	0.454917	-0.430671	0.887475	-0.0306102	0.97 [0.63 - 1.48]
GSE4922-GPL96	Breast cancer		Disease Free Survival	Uppsala (1987-1989)	249	-	0.431705	0.561868	0.115207	1.12 [0.76 - 1.66]
GSE2990	Breast cancer		Relapse Free Survival	Uppsala, Oxford	62	-	-1.36394	0.61683	-0.147222	0.86 [0.48 - 1.54]
GSE2990	Breast cancer		Distant Metastasis Free Survival	Uppsala, Oxford	125	-	-1.451	0.697863	-0.116862	0.89 [0.49 - 1.61]
GSE2990	Breast cancer		Relapse Free Survival	Uppsala, Oxford	125	0.103305	-1.01433	0.77866	-0.0660905	0.94 [0.59 - 1.48]
GSE2990	Breast cancer		Relapse Free Survival	Uppsala, Oxford	62	-	0.624519	0.812322	-0.139805	0.87 [0.27 - 2.76]
GSE2990	Breast cancer		Distant Metastasis Free Survival	Uppsala, Oxford	54	-	-1.80077	0.536202	-0.226278	0.80 [0.39 - 1.63]
GSE2990	Breast cancer		Distant Metastasis Free Survival	Uppsala, Oxford	125	0.469733	1.35915	0.421911	0.886458	2.43 [0.28 - 21.11]
GSE2990	Breast cancer		Relapse Free Survival	Uppsala, Oxford	125	-	0.716513	0.808643	0.220766	1.25 [0.21 - 7.44]
GSE2990	Breast cancer		Distant Metastasis Free Survival	Uppsala, Oxford	54	-	1.72306	0.873433	-0.114047	0.89 [0.22 - 3.63]
GSE7390	Breast cancer		Overall Survival	Uppsala, Oxford, Stockholm, IGR, GUYT, CRH (1980-1998)	198	0.121494	-0.839205	0.226341	-0.133524	0.88 [0.70 - 1.09]
GSE7390	Breast cancer		Distant Metastasis Free Survival	Uppsala, Oxford, Stockholm, IGR, GUYT, CRH (1980-1998)	198	-	0.600443	0.850834	0.0170532	1.02 [0.85 - 1.22]
GSE7390	Breast cancer		Overall Survival	Uppsala, Oxford, Stockholm, IGR, GUYT, CRH (1980-1998)	198	-	0.564955	0.96009	0.00467804	1.00 [0.84 - 1.21]
GSE7390	Breast cancer		Relapse Free Survival	Uppsala, Oxford, Stockholm, IGR, GUYT, CRH (1980-1998)	198	-	-0.440372	0.367299	-0.0774222	0.93 [0.78 - 1.10]
GSE7390	Breast cancer		Relapse Free Survival	Uppsala, Oxford, Stockholm, IGR, GUYT, CRH (1980-1998)	198	-	-0.276044	0.650076	-0.0330165	0.97 [0.84 - 1.12]
GSE7390	Breast cancer		Distant Metastasis Free Survival	Uppsala, Oxford, Stockholm, IGR, GUYT, CRH (1980-1998)	198	0.21788	-0.747658	0.204655	-0.132156	0.88 [0.71 - 1.07]
GSE12945	Colorectal cancer		Disease Free Survival	Berlin	51	0.304126	-16.2274	0.154904	-1.54727	0.21 [0.03 - 1.79]
GSE12945	Colorectal cancer		Overall Survival	Berlin	62	0.216632	-1.48632	0.111395	-0.812956	0.44 [0.16 - 1.21]
GSE12945	Colorectal cancer		Disease Free Survival	Berlin	51	0.171114	-2.30848	0.081331	-5.45647	0.00 [0.00 - 1.97]
GSE12945	Colorectal cancer		Overall Survival	Berlin	62	0.440131	-1.44824	0.165159	-1.87145	0.15 [0.01 - 2.16]
GSE17536	Colorectal cancer		Disease Specific Survival	MCC	177	-	-0.438602	0.0728629	0.513631	1.67 [0.95 - 2.93]
GSE17536	Colorectal cancer		Overall Survival	MCC	177	-	-0.464086	0.864087	0.0215462	1.02 [0.80 - 1.31]
GSE17536	Colorectal cancer		Disease Specific Survival	MCC	177	-	-0.553988	0.428353	0.110753	1.12 [0.85 - 1.47]
GSE17536	Colorectal cancer		Overall Survival	MCC	177	-	-0.335682	0.231035	0.329449	1.39 [0.81 - 2.38]
GSE17536	Colorectal cancer		Disease Free Survival	MCC	145	0.00212818	-1.33784	0.479681	-0.137206	0.87 [0.60 - 1.28]
GSE17536	Colorectal cancer		Disease Free Survival	MCC	145	-	-0.44028	0.832301	-0.105851	0.90 [0.34 - 2.40]
GSE14333	Colorectal cancer		Disease Free Survival	Melbourne	226	0.107761	-0.851047	0.230472	-0.165758	0.85 [0.65 - 1.11]
GSE14333	Colorectal cancer		Disease Free Survival	Melbourne	226	-	-0.49046	0.576476	-0.0829036	0.92 [0.69 - 1.23]
GSE17537	Colorectal cancer		Disease Specific Survival	VMC	49	-	0.722707	0.748343	0.113285	1.12 [0.56 - 2.24]
GSE17537	Colorectal cancer		Overall Survival	VMC	55	0.33542	0.972005	0.704135	0.0961675	1.10 [0.67 - 1.81]
GSE17537	Colorectal cancer		Overall Survival	VMC	55	-	15.2877	0.893685	0.077835	1.08 [0.35 - 3.39]
GSE17537	Colorectal cancer		Disease Free Survival	VMC	55	-	-0.731887	0.552902	-0.453664	0.64 [0.14 - 2.84]
GSE17537	Colorectal cancer		Disease Specific Survival	VMC	49	-	15.3584	0.391922	0.691785	2.00 [0.41 - 9.73]
GSE17537	Colorectal cancer		Disease Free Survival	VMC	55	0.334662	-1.23721	0.188496	-0.431707	0.65 [0.34 - 1.24]
GSE22138	Eye cancer	Uveal melanoma	Distant Metastasis Free Survival	BRCIC	63	-	-0.746515	0.844147	-0.0392081	0.96 [0.65 - 1.42]
GSE22138	Eye cancer	Uveal melanoma	Distant Metastasis Free Survival	BRCIC	63	0.107588	-0.970716	0.148597	-7.079	0.00 [0.00 - 12.51]
GSE2837	Head and neck cancer	Squamous cell carcinoma	Relapse Free Survival	VUMC, VAMC, UTMDACC (1992-2005)	28	-	1.05681	0.357221	0.254123	1.29 [0.75 - 2.21]
GSE2837	Head and neck cancer	Squamous cell carcinoma	Relapse Free Survival	VUMC, VAMC, UTMDACC (1992-2005)	28	-	-0.913687	0.968648	0.0893119	1.09 [0.01 - 93.97]
jacob-00182-CANDF	Lung cancer	Adenocarcinoma	Overall Survival	CAN/DF	82	-	0.556652	0.66758	0.111884	1.12 [0.67 - 1.86]
jacob-00182-CANDF	Lung cancer	Adenocarcinoma	Overall Survival	CAN/DF	82	-	-0.341307	0.624133	0.202288	1.22 [0.55 - 2.75]

jacob-00182-HLM	Lung cancer	Adenocarcinoma	Overall Survival	HLM	79	-	0.471047	0.960589	0.010045	1.01 [0.68 - 1.50]
jacob-00182-HLM	Lung cancer	Adenocarcinoma	Overall Survival	HLM	79	-	-0.454799	0.860853	-0.0611694	0.94 [0.47 - 1.86]
jacob-00182-MSK	Lung cancer	Adenocarcinoma	Overall Survival	MSK	104	0.0908584	-1.18713	0.29472	-0.420624	0.66 [0.30 - 1.44]
jacob-00182-MSK	Lung cancer	Adenocarcinoma	Overall Survival	MSK	104	0.0302494	-1.12163	0.0174422	-0.724217	0.48 [0.27 - 0.88]
GSE13213	Lung cancer	Adenocarcinoma	Overall Survival	Nagoya (1995-1999, 2002-2004)	117	0.0651012	-1.00806	0.122391	-0.179365	0.84 [0.67 - 1.05]
GSE13213	Lung cancer	Adenocarcinoma	Overall Survival	Nagoya (1995-1999, 2002-2004)	117	0.0217806	-0.933498	0.0202096	-0.249642	0.78 [0.63 - 0.96]
GSE31210	Lung cancer	Adenocarcinoma	Overall Survival	NCCRI	204	0.000246913	-1.53366	7.17E-05	-0.75405	0.47 [0.32 - 0.68]
GSE31210	Lung cancer	Adenocarcinoma	Relapse Free Survival	NCCRI	204	1.16E-06	-1.42107	2.07E-07	-0.796492	0.45 [0.33 - 0.61]
GSE31210	Lung cancer	Adenocarcinoma	Overall Survival	NCCRI	204	0.0926995	-0.992353	0.0124348	-0.679971	0.51 [0.30 - 0.86]
GSE31210	Lung cancer	Adenocarcinoma	Relapse Free Survival	NCCRI	204	6.22E-05	-1.28275	0.000108688	-0.786384	0.46 [0.31 - 0.68]
jacob-00182-UM	Lung cancer	Adenocarcinoma	Overall Survival	UM	178	0.24635	-0.469638	0.363868	-0.14732	0.86 [0.63 - 1.19]
jacob-00182-UM	Lung cancer	Adenocarcinoma	Overall Survival	UM	178	0.0226025	-0.654766	0.00822172	-0.606555	0.55 [0.35 - 0.85]
GSE3141	Lung cancer	NSCLC	Overall Survival	Duke	111	-	-0.462839	0.276982	-0.182875	0.83 [0.60 - 1.16]
GSE3141	Lung cancer	NSCLC	Overall Survival	Duke	111	0.444559	-0.692436	0.992823	-0.00126138	1.00 [0.76 - 1.31]
GSE14814	Lung cancer	NSCLC	Overall Survival	JRB.10	90	-	-0.973885	0.846501	0.0620714	1.06 [0.57 - 1.99]
GSE14814	Lung cancer	NSCLC	Disease Specific Survival	JRB.10	90	-	0.748914	0.654564	0.161335	1.18 [0.58 - 2.38]
GSE14814	Lung cancer	NSCLC	Overall Survival	JRB.10	90	0.130078	-0.993477	0.138763	-1.80607	0.16 [0.02 - 1.80]
GSE14814	Lung cancer	NSCLC	Disease Specific Survival	JRB.10	90	-	-0.744765	0.579293	-0.758669	0.47 [0.03 - 6.84]
GSE4716-GPL3696	Lung cancer	NSCLC	Overall Survival	Nagoya (1995-1996)	50	-	1.04649	0.645203	0.180444	1.20 [0.56 - 2.58]
GSE8894	Lung cancer	NSCLC	Relapse Free Survival	Seoul (1995-2005)	138	0.22671	-0.591484	0.124821	-0.140809	0.87 [0.73 - 1.04]
GSE8894	Lung cancer	NSCLC	Relapse Free Survival	Seoul (1995-2005)	138	0.063268	-0.871131	0.282915	-0.395141	0.67 [0.33 - 1.39]
GSE4573	Lung cancer	Squamous cell carcinoma	Overall Survival	Michigan (1991-2002)	129	-	-0.621606	0.435334	-0.173957	0.84 [0.54 - 1.30]
GSE4573	Lung cancer	Squamous cell carcinoma	Overall Survival	Michigan (1991-2002)	129	-	-0.427289	0.681739	-0.117783	0.89 [0.51 - 1.56]
GSE17710	Lung cancer	Squamous cell carcinoma	Overall Survival	UNC	56	0.386106	0.870721	0.31722	0.211889	1.24 [0.82 - 1.87]
GSE17710	Lung cancer	Squamous cell carcinoma	Relapse Free Survival	UNC	56	-	0.614579	0.447699	0.257882	1.29 [0.67 - 2.52]
GSE17710	Lung cancer	Squamous cell carcinoma	Relapse Free Survival	UNC	56	0.186888	0.863808	0.0785243	0.351716	1.42 [0.96 - 2.10]
GSE17710	Lung cancer	Squamous cell carcinoma	Overall Survival	UNC	56	-	0.575813	0.643379	0.163709	1.18 [0.59 - 2.36]
GSE17710	Lung cancer	Squamous cell carcinoma	Relapse Free Survival	UNC	56	0.215829	0.951698	0.153204	0.289062	1.34 [0.90 - 1.99]
GSE17710	Lung cancer	Squamous cell carcinoma	Overall Survival	UNC	56	0.381392	0.769709	0.166865	0.30022	1.35 [0.88 - 2.07]
GSE9891	Ovarian cancer		Overall Survival	AOCS, RBH, WH, NKI-AVL (1992-2006)	278	-	0.423163	0.528535	-0.171583	0.84 [0.49 - 1.44]
GSE9891	Ovarian cancer		Overall Survival	AOCS, RBH, WH, NKI-AVL (1992-2006)	278	-	0.315532	0.522985	0.042694	1.04 [0.92 - 1.19]
DUKE-OC	Ovarian cancer		Overall Survival	Duke	133	0.337722	0.734462	0.663045	-0.0386303	0.96 [0.81 - 1.14]
DUKE-OC	Ovarian cancer		Overall Survival	Duke	133	0.0729159	0.737623	0.283062	0.496944	1.64 [0.66 - 4.07]
GSE8841	Ovarian cancer		Overall Survival	Milan (1992-	81	0.262581	1.30847	0.0797566	0.926363	2.53 [0.90 - 7.12]
GSE26712	Ovarian cancer		Disease Free Survival	MSKCC (1990-2003)	185	0.147076	-0.577698	0.388562	-0.324856	0.72 [0.35 - 1.51]
GSE26712	Ovarian cancer		Disease Free Survival	MSKCC (1990-2003)	185	-	-0.30685	0.567359	-0.0556662	0.95 [0.78 - 1.14]
GSE26712	Ovarian cancer		Overall Survival	MSKCC (1990-2003)	185	-	0.459293	0.951766	-0.00616612	0.99 [0.81 - 1.21]
GSE26712	Ovarian cancer		Overall Survival	MSKCC (1990-2003)	185	-	-0.339689	0.28772	-0.465104	0.63 [0.27 - 1.48]
GSE17260	Ovarian cancer		Progression Free Survival	Niigata (1997-2008)	110	0.00579449	-1.01623	0.168161	-0.152591	0.86 [0.69 - 1.07]
GSE17260	Ovarian cancer		Overall Survival	Niigata (1997-2008)	110	0.0517887	-0.882568	0.183924	-0.201313	0.82 [0.61 - 1.10]
GSE17260	Ovarian cancer		Overall Survival	Niigata (1997-2008)	110	0.0626942	-0.946651	0.181943	-0.204744	0.81 [0.60 - 1.10]
GSE17260	Ovarian cancer		Progression Free Survival	Niigata (1997-2008)	110	0.00369769	-0.872029	0.0820984	-0.198502	0.82 [0.66 - 1.03]
GSE14764	Ovarian cancer		Overall Survival	TOC	80	-	0.732848	0.344974	0.306199	1.36 [0.72 - 2.56]
GSE14764	Ovarian cancer		Overall Survival	TOC	80	-	-0.861283	0.423531	-0.146981	0.86 [0.60 - 1.24]
GSE19234	Skin cancer	Melanoma	Overall Survival	NYU	38	0.220741	-1.07611	0.0383605	-0.522893	0.59 [0.36 - 0.97]
GSE19234	Skin cancer	Melanoma	Overall Survival	NYU	38	-	-1.37226	0.609644	-0.153119	0.86 [0.48 - 1.54]
GSE30929	Soft tissue cancer	Liposarcoma	Distant Recurrence Free Survival	MSKCC (1993-2008)	140	0.0435859	-1.01188	0.857574	0.0442297	1.05 [0.64 - 1.69]
GSE30929	Soft tissue cancer	Liposarcoma	Distant Recurrence Free Survival	MSKCC (1993-2008)	140	0.205514	-0.701146	0.665412	0.45687	1.58 [0.20 - 12.52]

Supplementary Table 2. The correlations between TOX and immune infiltration levels in different cancer types.

cancer	variable	partial.cor	p
ACC	Purity	-0.290882042	0.011927411
ACC	B Cell	0.246443994	0.035569818
ACC	CD8+ T Cell	0.280626719	0.016179968
ACC	CD4+ T Cell	0.12219141	0.303080521
ACC	Macrophage	0.276443604	0.017908432
ACC	Neutrophil	0.166506938	0.159151399
ACC	Dendritic Cell	0.289584583	0.012955072
BLCA	Purity	-0.388781488	9.20E-15
BLCA	B Cell	0.163839236	0.001736314
BLCA	CD8+ T Cell	0.149587964	0.004128777
BLCA	CD4+ T Cell	0.3217785	3.08E-10
BLCA	Macrophage	0.128450033	0.014057205
BLCA	Neutrophil	0.378254132	8.60E-14
BLCA	Dendritic Cell	0.310092135	1.41E-09
BRCA	Purity	-0.512089465	1.30E-67
BRCA	B Cell	0.434571095	2.84E-46
BRCA	CD8+ T Cell	0.537524938	3.60E-74
BRCA	CD4+ T Cell	0.533792625	5.86E-72
BRCA	Macrophage	0.093855385	0.003225462
BRCA	Neutrophil	0.49581328	3.76E-60
BRCA	Dendritic Cell	0.571131718	1.98E-83
BRCA-Basal	Purity	-0.423684968	5.64E-07
BRCA-Basal	B Cell	0.376095188	1.67E-05
BRCA-Basal	CD8+ T Cell	0.365413448	3.24E-05
BRCA-Basal	CD4+ T Cell	0.410989236	2.57E-06
BRCA-Basal	Macrophage	-0.00835129	0.925756329
BRCA-Basal	Neutrophil	0.246756188	0.009356318
BRCA-Basal	Dendritic Cell	0.427106304	2.15E-06
BRCA-Her2	Purity	-0.543743014	8.56E-06
BRCA-Her2	B Cell	0.343925727	0.008207482
BRCA-Her2	CD8+ T Cell	0.682190332	5.11E-09
BRCA-Her2	CD4+ T Cell	0.588820969	1.16E-06
BRCA-Her2	Macrophage	-0.004158393	0.975285415
BRCA-Her2	Neutrophil	0.556431253	5.75E-06
BRCA-Her2	Dendritic Cell	0.604739356	7.97E-07
BRCA-Luminal	Purity	-0.52902585	1.06E-40
BRCA-Luminal	B Cell	0.420769652	1.26E-24
BRCA-Luminal	CD8+ T Cell	0.645073908	1.65E-64
BRCA-Luminal	CD4+ T Cell	0.562201997	5.42E-46
BRCA-Luminal	Macrophage	0.211994741	6.33E-07
BRCA-Luminal	Neutrophil	0.501175725	2.64E-35
BRCA-Luminal	Dendritic Cell	0.567959279	5.09E-47
CESC	Purity	-0.2804119	2.03E-06
CESC	B Cell	0.228329844	0.000126206
CESC	CD8+ T Cell	0.450428849	4.28E-15
CESC	CD4+ T Cell	0.226369367	0.000144729
CESC	Macrophage	0.170540246	0.004421647
CESC	Neutrophil	0.356325482	1.03E-09
CESC	Dendritic Cell	0.356071115	1.13E-09
CHOL	Purity	-0.119850673	0.486277561
CHOL	B Cell	0.438017092	0.008496026
CHOL	CD8+ T Cell	0.358437429	0.034484824
CHOL	CD4+ T Cell	0.344634436	0.042619057
CHOL	Macrophage	0.475554049	0.003886602

CHOL	Neutrophil	0.309152237	0.070745983
CHOL	Dendritic Cell	0.485853671	0.003087657
COAD	Purity	-0.267608843	4.19E-08
COAD	B Cell	0.210181327	2.05E-05
COAD	CD8+ T Cell	0.224893202	4.73E-06
COAD	CD4+ T Cell	0.061966553	0.21506846
COAD	Macrophage	0.053767155	0.280972211
COAD	Neutrophil	0.096069522	0.054576399
COAD	Dendritic Cell	0.207419744	2.77E-05
DLBC	Purity	0.010456775	0.947597369
DLBC	B Cell	0.320385077	0.194911901
DLBC	CD8+ T Cell	-0.111284649	0.631057032
DLBC	CD4+ T Cell	0.017802949	0.938946981
DLBC	Macrophage	0.263132382	0.249140464
DLBC	Neutrophil	-0.168150145	0.466252779
DLBC	Dendritic Cell	0.007741732	0.97343135
ESCA	Purity	-0.188852622	0.010893309
ESCA	B Cell	0.253965178	0.00060261
ESCA	CD8+ T Cell	0.055476683	0.459492748
ESCA	CD4+ T Cell	0.283782995	0.000118309
ESCA	Macrophage	0.262240009	0.000376184
ESCA	Neutrophil	0.173451097	0.019882562
ESCA	Dendritic Cell	0.190538759	0.010405566
GBM	Purity	0.076193116	0.119413675
GBM	B Cell	0.060381995	0.21797138
GBM	CD8+ T Cell	0.092345129	0.059243855
GBM	CD4+ T Cell	0.094873218	0.052592373
GBM	Macrophage	0.142873695	0.003418704
GBM	Neutrophil	0.156007514	0.001375983
GBM	Dendritic Cell	-0.111521527	0.022586774
HNSC	Purity	-0.249495775	1.97E-08
HNSC	B Cell	0.269911998	2.17E-09
HNSC	CD8+ T Cell	0.284947945	2.64E-10
HNSC	CD4+ T Cell	0.391232062	5.26E-19
HNSC	Macrophage	0.381199052	3.75E-18
HNSC	Neutrophil	0.404597579	2.72E-20
HNSC	Dendritic Cell	0.473641907	2.54E-28
HNSC-HPVpos	Purity	-0.37564447	0.000263844
HNSC-HPVpos	B Cell	0.414026788	0.000148841
HNSC-HPVpos	CD8+ T Cell	0.403713406	0.000247102
HNSC-HPVpos	CD4+ T Cell	0.430879078	5.32E-05
HNSC-HPVpos	Macrophage	0.535312295	1.09E-07
HNSC-HPVpos	Neutrophil	0.487616444	2.21E-06
HNSC-HPVpos	Dendritic Cell	0.581123382	8.41E-09
HNSC-HPVneg	Purity	-0.227477206	4.20E-06
HNSC-HPVneg	B Cell	0.25108802	4.44E-07
HNSC-HPVneg	CD8+ T Cell	0.263492089	1.15E-07
HNSC-HPVneg	CD4+ T Cell	0.388619506	1.09E-15
HNSC-HPVneg	Macrophage	0.34252826	2.75E-12
HNSC-HPVneg	Neutrophil	0.387653898	1.81E-15
HNSC-HPVneg	Dendritic Cell	0.458183357	6.01E-22
KICH	Purity	-0.390222804	0.001199243
KICH	B Cell	0.284556917	0.021597443
KICH	CD8+ T Cell	-0.024987041	0.84337842
KICH	CD4+ T Cell	0.078364294	0.534935476
KICH	Macrophage	-0.017457266	0.890221019

KICH	Neutrophil	0.163542172	0.193012254
KICH	Dendritic Cell	0.320763089	0.009184029
KIRC	Purity	-0.270532022	3.43E-09
KIRC	B Cell	0.482873384	3.46E-28
KIRC	CD8+ T Cell	0.620848815	4.92E-48
KIRC	CD4+ T Cell	0.196098326	2.28E-05
KIRC	Macrophage	0.243254869	1.86E-07
KIRC	Neutrophil	0.422448362	3.00E-21
KIRC	Dendritic Cell	0.578019422	6.47E-42
KIRP	Purity	-0.133055187	0.03231526
KIRP	B Cell	0.372514535	7.56E-10
KIRP	CD8+ T Cell	0.323133448	1.11E-07
KIRP	CD4+ T Cell	0.157568707	0.011261093
KIRP	Macrophage	0.050623194	0.426432514
KIRP	Neutrophil	0.209214853	0.000720464
KIRP	Dendritic Cell	0.233622297	0.000161949
LGG	Purity	0.263556135	4.71E-09
LGG	B Cell	-0.228440841	4.46E-07
LGG	CD8+ T Cell	-0.174247607	0.000128681
LGG	CD4+ T Cell	-0.183306346	5.75E-05
LGG	Macrophage	-0.27468609	1.24E-09
LGG	Neutrophil	-0.279778375	5.44E-10
LGG	Dendritic Cell	-0.236718389	1.74E-07
LIHC	Purity	-0.440307653	7.69E-18
LIHC	B Cell	0.352910388	1.59E-11
LIHC	CD8+ T Cell	0.456262943	5.48E-19
LIHC	CD4+ T Cell	0.139616139	0.00952039
LIHC	Macrophage	0.273216995	2.98E-07
LIHC	Neutrophil	0.28246016	9.48E-08
LIHC	Dendritic Cell	0.420897536	4.97E-16
LUAD	Purity	-0.046099361	0.306517094
LUAD	B Cell	0.325670236	2.02E-13
LUAD	CD8+ T Cell	0.225450899	4.97E-07
LUAD	CD4+ T Cell	0.202462123	7.15E-06
LUAD	Macrophage	0.132025951	0.003581719
LUAD	Neutrophil	0.083772335	0.065834232
LUAD	Dendritic Cell	0.22342354	6.16E-07
LUSC	Purity	-0.21015379	3.58E-06
LUSC	B Cell	0.400900639	1.19E-19
LUSC	CD8+ T Cell	0.408295988	1.65E-20
LUSC	CD4+ T Cell	0.282954149	3.40E-10
LUSC	Macrophage	0.273840047	1.19E-09
LUSC	Neutrophil	0.278880278	5.95E-10
LUSC	Dendritic Cell	0.421235244	8.24E-22
MESO	Purity	-0.228565511	0.034283685
MESO	B Cell	0.519300173	4.16E-07
MESO	CD8+ T Cell	0.195631675	0.074520704
MESO	CD4+ T Cell	0.307698918	0.004409499
MESO	Macrophage	-0.043369974	0.695264513
MESO	Neutrophil	0.004872051	0.964916953
MESO	Dendritic Cell	0.700798027	1.16E-13
OV	Purity	0.187474421	3.32E-05
OV	B Cell	-0.117673329	0.00987025
OV	CD8+ T Cell	0.021470263	0.638914143
OV	CD4+ T Cell	-0.060024439	0.189242007
OV	Macrophage	-0.128916591	0.004671266

OV	Neutrophil	-0.144304563	0.001524819
OV	Dendritic Cell	-0.060490714	0.185821567
PAAD	Purity	0.028608347	0.709494291
PAAD	B Cell	0.285021974	0.000157783
PAAD	CD8+ T Cell	0.291370305	0.000110432
PAAD	CD4+ T Cell	0.21350778	0.005316328
PAAD	Macrophage	0.335735512	7.15E-06
PAAD	Neutrophil	0.180899987	0.017894552
PAAD	Dendritic Cell	0.234008789	0.0020665
PCPG	Purity	0.016402295	0.832867794
PCPG	B Cell	0.15058622	0.052079282
PCPG	CD8+ T Cell	0.038674968	0.619737404
PCPG	CD4+ T Cell	0.161327923	0.037267567
PCPG	Macrophage	0.170741517	0.027849208
PCPG	Neutrophil	0.057453313	0.460822395
PCPG	Dendritic Cell	0.305757313	5.86E-05
PRAD	Purity	-0.243114702	5.03E-07
PRAD	B Cell	0.312383703	8.91E-11
PRAD	CD8+ T Cell	0.186215009	0.000133402
PRAD	CD4+ T Cell	0.217106388	8.95E-06
PRAD	Macrophage	0.171418405	0.000445132
PRAD	Neutrophil	0.251927591	2.05E-07
PRAD	Dendritic Cell	0.253906368	1.58E-07
READ	Purity	-0.183049433	0.030404739
READ	B Cell	0.238667637	0.004662829
READ	CD8+ T Cell	0.223437415	0.008192921
READ	CD4+ T Cell	-0.024224421	0.77712762
READ	Macrophage	-0.058304693	0.495381171
READ	Neutrophil	-0.043662432	0.611106538
READ	Dendritic Cell	0.246642536	0.0034228
SARC	Purity	-0.112414199	0.079065775
SARC	B Cell	0.377312677	1.67E-09
SARC	CD8+ T Cell	0.132313059	0.040550526
SARC	CD4+ T Cell	0.069820932	0.282348362
SARC	Macrophage	-0.105475659	0.106789838
SARC	Neutrophil	-0.013610014	0.833171572
SARC	Dendritic Cell	0.138363745	0.031781071
SKCM	Purity	-0.498123906	4.29E-30
SKCM	B Cell	0.289247994	4.40E-10
SKCM	CD8+ T Cell	0.503267934	1.68E-29
SKCM	CD4+ T Cell	0.384835127	3.42E-17
SKCM	Macrophage	0.31341058	8.80E-12
SKCM	Neutrophil	0.498745286	8.44E-30
SKCM	Dendritic Cell	0.499622837	1.54E-29
SKCM-Primary	Purity	-0.442082916	2.94E-06
SKCM-Primary	B Cell	0.090264101	0.369353081
SKCM-Primary	CD8+ T Cell	0.407923429	2.29E-05
SKCM-Primary	CD4+ T Cell	0.390204389	5.49E-05
SKCM-Primary	Macrophage	0.117352631	0.24250732
SKCM-Primary	Neutrophil	0.267631289	0.007103388
SKCM-Primary	Dendritic Cell	0.275880829	0.005230789
SKCM-Metastasis	Purity	-0.528039879	7.02E-27
SKCM-Metastasis	B Cell	0.283044374	8.52E-08
SKCM-Metastasis	CD8+ T Cell	0.478726521	1.19E-20
SKCM-Metastasis	CD4+ T Cell	0.326183435	5.71E-10
SKCM-Metastasis	Macrophage	0.266750553	3.94E-07

SKCM-Metastasis	Neutrophil	0.49766503	2.31E-23
SKCM-Metastasis	Dendritic Cell	0.505555345	1.02E-23
STAD	Purity	-0.118839836	0.020492837
STAD	B Cell	0.298542393	4.94E-09
STAD	CD8+ T Cell	0.169357676	0.001074367
STAD	CD4+ T Cell	0.425809021	1.49E-17
STAD	Macrophage	0.3535631	2.46E-12
STAD	Neutrophil	0.120489218	0.020264296
STAD	Dendritic Cell	0.268506954	1.51E-07
TGCT	Purity	-0.095775904	0.246884899
TGCT	B Cell	0.294394912	0.000295257
TGCT	CD8+ T Cell	0.204183803	0.013114121
TGCT	CD4+ T Cell	0.262005665	0.00140019
TGCT	Macrophage	0.220049363	0.007404351
TGCT	Neutrophil	0.256845275	0.001687435
TGCT	Dendritic Cell	0.169236064	0.041145151
THCA	Purity	-0.070525522	0.119350941
THCA	B Cell	0.238326319	1.19E-07
THCA	CD8+ T Cell	0.447100053	2.62E-25
THCA	CD4+ T Cell	0.211072272	2.55E-06
THCA	Macrophage	0.291445057	5.21E-11
THCA	Neutrophil	0.506290519	3.95E-33
THCA	Dendritic Cell	0.559927843	2.27E-41
THYM	Purity	-0.116666327	0.212327538
THYM	B Cell	0.734431417	1.43E-20
THYM	CD8+ T Cell	0.60831737	7.03E-13
THYM	CD4+ T Cell	0.806638501	1.20E-26
THYM	Macrophage	0.525574751	1.92E-09
THYM	Neutrophil	-0.107501621	0.25492996
THYM	Dendritic Cell	0.897528091	1.24E-41
UCEC	Purity	-0.181631028	0.001765357
UCEC	B Cell	0.210200056	0.000320432
UCEC	CD8+ T Cell	0.128495634	0.028960756
UCEC	CD4+ T Cell	0.195489682	0.000799816
UCEC	Macrophage	0.248739472	1.71E-05
UCEC	Neutrophil	0.136656273	0.019275047
UCEC	Dendritic Cell	0.150888504	0.009819414
UCS	Purity	-0.024063764	0.862872223
UCS	B Cell	0.147556285	0.291701737
UCS	CD8+ T Cell	0.087264421	0.534380617
UCS	CD4+ T Cell	0.145551551	0.29837952
UCS	Macrophage	-0.029947936	0.831425939
UCS	Neutrophil	0.203167504	0.144543402
UCS	Dendritic Cell	0.143048282	0.306855876
UVM	Purity	0.16890095	0.139340231
UVM	B Cell	-0.22079624	0.05696217
UVM	CD8+ T Cell	0.155955759	0.175600447
UVM	CD4+ T Cell	0.085826824	0.461011506
UVM	Macrophage	0.224813957	0.074098711
UVM	Neutrophil	0.012271445	0.915642514
UVM	Dendritic Cell	-0.018267355	0.877231838

Supplementary Table 3. The detailed SCNA (somatic copy number alterations) analysis of TOX for various cancers.

cancer	variable	cna_level	p
ACC	B Cell	Arm-level Deletion	0.317467885
ACC	B Cell	Diploid/Normal	1
ACC	B Cell	Arm-level Gain	0.25620585
ACC	CD8+ T Cell	Arm-level Deletion	0.022628271
ACC	CD8+ T Cell	Diploid/Normal	1
ACC	CD8+ T Cell	Arm-level Gain	0.150142937
ACC	CD4+ T Cell	Arm-level Deletion	0.691024701
ACC	CD4+ T Cell	Diploid/Normal	1
ACC	CD4+ T Cell	Arm-level Gain	0.531647595
ACC	Macrophage	Arm-level Deletion	0.027403288
ACC	Macrophage	Diploid/Normal	1
ACC	Macrophage	Arm-level Gain	0.362707983
ACC	Neutrophil	Arm-level Deletion	0.039044481
ACC	Neutrophil	Diploid/Normal	1
ACC	Neutrophil	Arm-level Gain	0.143993681
ACC	Dendritic Cell	Arm-level Deletion	0.016726061
ACC	Dendritic Cell	Diploid/Normal	1
ACC	Dendritic Cell	Arm-level Gain	0.060800445
BLCA	B Cell	Deep Deletion	0.444192645
BLCA	B Cell	Arm-level Deletion	0.021686487
BLCA	B Cell	Diploid/Normal	1
BLCA	B Cell	Arm-level Gain	0.306140006
BLCA	B Cell	High Amplication	0.630679678
BLCA	CD8+ T Cell	Deep Deletion	0.294178078
BLCA	CD8+ T Cell	Arm-level Deletion	0.163164434
BLCA	CD8+ T Cell	Diploid/Normal	1
BLCA	CD8+ T Cell	Arm-level Gain	0.834158379
BLCA	CD8+ T Cell	High Amplication	0.254954877
BLCA	CD4+ T Cell	Deep Deletion	0.179635444
BLCA	CD4+ T Cell	Arm-level Deletion	0.653026117
BLCA	CD4+ T Cell	Diploid/Normal	1
BLCA	CD4+ T Cell	Arm-level Gain	0.101766056
BLCA	CD4+ T Cell	High Amplication	0.542792954
BLCA	Macrophage	Deep Deletion	0.920702909
BLCA	Macrophage	Arm-level Deletion	0.212787276
BLCA	Macrophage	Diploid/Normal	1
BLCA	Macrophage	Arm-level Gain	0.744384801
BLCA	Macrophage	High Amplication	0.215235112
BLCA	Neutrophil	Deep Deletion	0.007969667
BLCA	Neutrophil	Arm-level Deletion	0.510409206
BLCA	Neutrophil	Diploid/Normal	1
BLCA	Neutrophil	Arm-level Gain	0.282446331
BLCA	Neutrophil	High Amplication	0.576396035
BLCA	Dendritic Cell	Deep Deletion	0.075818012
BLCA	Dendritic Cell	Arm-level Deletion	0.686958816
BLCA	Dendritic Cell	Diploid/Normal	1
BLCA	Dendritic Cell	Arm-level Gain	0.354627526
BLCA	Dendritic Cell	High Amplication	0.678185441
BRCA	B Cell	Deep Deletion	1
BRCA	B Cell	Arm-level Deletion	0.33789253
BRCA	B Cell	Diploid/Normal	1
BRCA	B Cell	Arm-level Gain	0.363551684
BRCA	B Cell	High Amplication	0.14792963
BRCA	CD8+ T Cell	Deep Deletion	1
BRCA	CD8+ T Cell	Arm-level Deletion	0.922551944
BRCA	CD8+ T Cell	Diploid/Normal	1

BRCA	CD8+ T Cell	Arm-level Gain	0.305905831
BRCA	CD8+ T Cell	High Amplication	0.06853451
BRCA	CD4+ T Cell	Deep Deletion	1
BRCA	CD4+ T Cell	Arm-level Deletion	0.302137703
BRCA	CD4+ T Cell	Diploid/Normal	1
BRCA	CD4+ T Cell	Arm-level Gain	0.053545426
BRCA	CD4+ T Cell	High Amplication	0.000278395
BRCA	Macrophage	Deep Deletion	1
BRCA	Macrophage	Arm-level Deletion	0.292974149
BRCA	Macrophage	Diploid/Normal	1
BRCA	Macrophage	Arm-level Gain	0.018065447
BRCA	Macrophage	High Amplication	0.029759762
BRCA	Neutrophil	Deep Deletion	1
BRCA	Neutrophil	Arm-level Deletion	0.975942885
BRCA	Neutrophil	Diploid/Normal	1
BRCA	Neutrophil	Arm-level Gain	0.587461736
BRCA	Neutrophil	High Amplication	0.01775741
BRCA	Dendritic Cell	Deep Deletion	1
BRCA	Dendritic Cell	Arm-level Deletion	0.681791488
BRCA	Dendritic Cell	Diploid/Normal	1
BRCA	Dendritic Cell	Arm-level Gain	0.47599111
BRCA	Dendritic Cell	High Amplication	0.039511548
BRCA-Basal	B Cell	Arm-level Deletion	0.08501973
BRCA-Basal	B Cell	Diploid/Normal	1
BRCA-Basal	B Cell	Arm-level Gain	0.112044998
BRCA-Basal	B Cell	High Amplication	0.311694434
BRCA-Basal	CD8+ T Cell	Arm-level Deletion	0.011968175
BRCA-Basal	CD8+ T Cell	Diploid/Normal	1
BRCA-Basal	CD8+ T Cell	Arm-level Gain	0.255739923
BRCA-Basal	CD8+ T Cell	High Amplication	0.264808039
BRCA-Basal	CD4+ T Cell	Arm-level Deletion	0.099028477
BRCA-Basal	CD4+ T Cell	Diploid/Normal	1
BRCA-Basal	CD4+ T Cell	Arm-level Gain	0.562043528
BRCA-Basal	CD4+ T Cell	High Amplication	0.09235737
BRCA-Basal	Macrophage	Arm-level Deletion	0.457784793
BRCA-Basal	Macrophage	Diploid/Normal	1
BRCA-Basal	Macrophage	Arm-level Gain	0.82347073
BRCA-Basal	Macrophage	High Amplication	0.055249588
BRCA-Basal	Neutrophil	Arm-level Deletion	0.187497032
BRCA-Basal	Neutrophil	Diploid/Normal	1
BRCA-Basal	Neutrophil	Arm-level Gain	0.691497412
BRCA-Basal	Neutrophil	High Amplication	0.11910873
BRCA-Basal	Dendritic Cell	Arm-level Deletion	0.068117727
BRCA-Basal	Dendritic Cell	Diploid/Normal	1
BRCA-Basal	Dendritic Cell	Arm-level Gain	0.365199119
BRCA-Basal	Dendritic Cell	High Amplication	0.14148059
BRCA-Her2	B Cell	Deep Deletion	1
BRCA-Her2	B Cell	Arm-level Deletion	0.580772795
BRCA-Her2	B Cell	Diploid/Normal	1
BRCA-Her2	B Cell	Arm-level Gain	0.375551366
BRCA-Her2	B Cell	High Amplication	0.618811926
BRCA-Her2	CD8+ T Cell	Deep Deletion	1
BRCA-Her2	CD8+ T Cell	Arm-level Deletion	0.344292419
BRCA-Her2	CD8+ T Cell	Diploid/Normal	1
BRCA-Her2	CD8+ T Cell	Arm-level Gain	0.393555223
BRCA-Her2	CD8+ T Cell	High Amplication	0.679314277
BRCA-Her2	CD4+ T Cell	Deep Deletion	1
BRCA-Her2	CD4+ T Cell	Arm-level Deletion	0.848344344

BRCA-Her2	CD4+ T Cell	Diploid/Normal	1
BRCA-Her2	CD4+ T Cell	Arm-level Gain	0.864621341
BRCA-Her2	CD4+ T Cell	High Amplication	0.804495553
BRCA-Her2	Macrophage	Deep Deletion	1
BRCA-Her2	Macrophage	Arm-level Deletion	0.192320083
BRCA-Her2	Macrophage	Diploid/Normal	1
BRCA-Her2	Macrophage	Arm-level Gain	0.079369861
BRCA-Her2	Macrophage	High Amplication	0.674213933
BRCA-Her2	Neutrophil	Deep Deletion	1
BRCA-Her2	Neutrophil	Arm-level Deletion	0.867753707
BRCA-Her2	Neutrophil	Diploid/Normal	1
BRCA-Her2	Neutrophil	Arm-level Gain	0.926711032
BRCA-Her2	Neutrophil	High Amplication	0.985877254
BRCA-Her2	Dendritic Cell	Deep Deletion	1
BRCA-Her2	Dendritic Cell	Arm-level Deletion	0.819750747
BRCA-Her2	Dendritic Cell	Diploid/Normal	1
BRCA-Her2	Dendritic Cell	Arm-level Gain	0.451359402
BRCA-Her2	Dendritic Cell	High Amplication	0.914010631
BRCA-Luminal	B Cell	Arm-level Deletion	0.528174928
BRCA-Luminal	B Cell	Diploid/Normal	1
BRCA-Luminal	B Cell	Arm-level Gain	0.37065885
BRCA-Luminal	B Cell	High Amplication	0.043434506
BRCA-Luminal	CD8+ T Cell	Arm-level Deletion	0.287064757
BRCA-Luminal	CD8+ T Cell	Diploid/Normal	1
BRCA-Luminal	CD8+ T Cell	Arm-level Gain	0.415521697
BRCA-Luminal	CD8+ T Cell	High Amplication	0.008054574
BRCA-Luminal	CD4+ T Cell	Arm-level Deletion	0.947696126
BRCA-Luminal	CD4+ T Cell	Diploid/Normal	1
BRCA-Luminal	CD4+ T Cell	Arm-level Gain	0.070163051
BRCA-Luminal	CD4+ T Cell	High Amplication	9.73E-05
BRCA-Luminal	Macrophage	Arm-level Deletion	0.242485494
BRCA-Luminal	Macrophage	Diploid/Normal	1
BRCA-Luminal	Macrophage	Arm-level Gain	0.060857064
BRCA-Luminal	Macrophage	High Amplication	0.288331141
BRCA-Luminal	Neutrophil	Arm-level Deletion	0.45678677
BRCA-Luminal	Neutrophil	Diploid/Normal	1
BRCA-Luminal	Neutrophil	Arm-level Gain	0.125376965
BRCA-Luminal	Neutrophil	High Amplication	0.004104564
BRCA-Luminal	Dendritic Cell	Arm-level Deletion	0.3946298
BRCA-Luminal	Dendritic Cell	Diploid/Normal	1
BRCA-Luminal	Dendritic Cell	Arm-level Gain	0.315389327
BRCA-Luminal	Dendritic Cell	High Amplication	0.011372023
CESC	B Cell	Arm-level Deletion	0.15571049
CESC	B Cell	Diploid/Normal	1
CESC	B Cell	Arm-level Gain	0.311580743
CESC	B Cell	High Amplication	0.704679925
CESC	CD8+ T Cell	Arm-level Deletion	0.412436117
CESC	CD8+ T Cell	Diploid/Normal	1
CESC	CD8+ T Cell	Arm-level Gain	0.18537661
CESC	CD8+ T Cell	High Amplication	0.626084534
CESC	CD4+ T Cell	Arm-level Deletion	0.680407062
CESC	CD4+ T Cell	Diploid/Normal	1
CESC	CD4+ T Cell	Arm-level Gain	0.646220184
CESC	CD4+ T Cell	High Amplication	0.448782419
CESC	Macrophage	Arm-level Deletion	0.758170071
CESC	Macrophage	Diploid/Normal	1
CESC	Macrophage	Arm-level Gain	0.677632363
CESC	Macrophage	High Amplication	0.521664155

CESC	Neutrophil	Arm-level Deletion	0.809123837
CESC	Neutrophil	Diploid/Normal	1
CESC	Neutrophil	Arm-level Gain	0.802619293
CESC	Neutrophil	High Amplication	0.798686947
CESC	Dendritic Cell	Arm-level Deletion	0.924416726
CESC	Dendritic Cell	Diploid/Normal	1
CESC	Dendritic Cell	Arm-level Gain	0.816697687
CESC	Dendritic Cell	High Amplication	0.685555674
CHOL	B Cell	Arm-level Deletion	0.562233099
CHOL	B Cell	Diploid/Normal	1
CHOL	B Cell	Arm-level Gain	0.554701308
CHOL	CD8+ T Cell	Arm-level Deletion	0.523274104
CHOL	CD8+ T Cell	Diploid/Normal	1
CHOL	CD8+ T Cell	Arm-level Gain	0.34072511
CHOL	CD4+ T Cell	Arm-level Deletion	0.936923777
CHOL	CD4+ T Cell	Diploid/Normal	1
CHOL	CD4+ T Cell	Arm-level Gain	0.995110894
CHOL	Macrophage	Arm-level Deletion	0.795841769
CHOL	Macrophage	Diploid/Normal	1
CHOL	Macrophage	Arm-level Gain	0.39659534
CHOL	Neutrophil	Arm-level Deletion	0.461480613
CHOL	Neutrophil	Diploid/Normal	1
CHOL	Neutrophil	Arm-level Gain	0.039481714
CHOL	Dendritic Cell	Arm-level Deletion	0.59135491
CHOL	Dendritic Cell	Diploid/Normal	1
CHOL	Dendritic Cell	Arm-level Gain	0.41775693
COAD	B Cell	Arm-level Deletion	0.047169032
COAD	B Cell	Diploid/Normal	1
COAD	B Cell	Arm-level Gain	0.000312569
COAD	B Cell	High Amplication	0.236959117
COAD	CD8+ T Cell	Arm-level Deletion	0.026617302
COAD	CD8+ T Cell	Diploid/Normal	1
COAD	CD8+ T Cell	Arm-level Gain	0.002340936
COAD	CD8+ T Cell	High Amplication	0.308106146
COAD	CD4+ T Cell	Arm-level Deletion	0.441369636
COAD	CD4+ T Cell	Diploid/Normal	1
COAD	CD4+ T Cell	Arm-level Gain	0.529715892
COAD	CD4+ T Cell	High Amplication	0.375699691
COAD	Macrophage	Arm-level Deletion	0.583695136
COAD	Macrophage	Diploid/Normal	1
COAD	Macrophage	Arm-level Gain	0.129638297
COAD	Macrophage	High Amplication	0.305176627
COAD	Neutrophil	Arm-level Deletion	0.221993705
COAD	Neutrophil	Diploid/Normal	1
COAD	Neutrophil	Arm-level Gain	0.360714101
COAD	Neutrophil	High Amplication	0.576649472
COAD	Dendritic Cell	Arm-level Deletion	0.145226203
COAD	Dendritic Cell	Diploid/Normal	1
COAD	Dendritic Cell	Arm-level Gain	0.035067408
COAD	Dendritic Cell	High Amplication	0.099808093
DLBC	B Cell	Deep Deletion	0.125781884
DLBC	B Cell	Arm-level Deletion	0.025051226
DLBC	B Cell	Diploid/Normal	1
DLBC	CD8+ T Cell	Deep Deletion	0.367329732
DLBC	CD8+ T Cell	Arm-level Deletion	0.463555477
DLBC	CD8+ T Cell	Diploid/Normal	1
DLBC	CD4+ T Cell	Deep Deletion	0.594578113
DLBC	CD4+ T Cell	Arm-level Deletion	0.855973044

DLBC	CD4+ T Cell	Diploid/Normal	1
DLBC	Macrophage	Deep Deletion	0.007975121
DLBC	Macrophage	Arm-level Deletion	0.531851274
DLBC	Macrophage	Diploid/Normal	1
DLBC	Neutrophil	Deep Deletion	0.545886116
DLBC	Neutrophil	Arm-level Deletion	0.693802235
DLBC	Neutrophil	Diploid/Normal	1
DLBC	Dendritic Cell	Deep Deletion	0.203879642
DLBC	Dendritic Cell	Arm-level Deletion	0.468169329
DLBC	Dendritic Cell	Diploid/Normal	1
ESCA	B Cell	Arm-level Deletion	0.371035693
ESCA	B Cell	Diploid/Normal	1
ESCA	B Cell	Arm-level Gain	0.1618897
ESCA	B Cell	High Amplication	0.402261238
ESCA	CD8+ T Cell	Arm-level Deletion	0.517070358
ESCA	CD8+ T Cell	Diploid/Normal	1
ESCA	CD8+ T Cell	Arm-level Gain	0.751636104
ESCA	CD8+ T Cell	High Amplication	0.445278909
ESCA	CD4+ T Cell	Arm-level Deletion	0.187859346
ESCA	CD4+ T Cell	Diploid/Normal	1
ESCA	CD4+ T Cell	Arm-level Gain	0.137419904
ESCA	CD4+ T Cell	High Amplication	0.434265522
ESCA	Macrophage	Arm-level Deletion	0.620708337
ESCA	Macrophage	Diploid/Normal	1
ESCA	Macrophage	Arm-level Gain	0.928533426
ESCA	Macrophage	High Amplication	0.53569308
ESCA	Neutrophil	Arm-level Deletion	0.00868741
ESCA	Neutrophil	Diploid/Normal	1
ESCA	Neutrophil	Arm-level Gain	0.115477742
ESCA	Neutrophil	High Amplication	0.979102305
ESCA	Dendritic Cell	Arm-level Deletion	0.013534341
ESCA	Dendritic Cell	Diploid/Normal	1
ESCA	Dendritic Cell	Arm-level Gain	0.010337827
ESCA	Dendritic Cell	High Amplication	0.693946952
GBM	B Cell	Arm-level Deletion	0.980568029
GBM	B Cell	Diploid/Normal	1
GBM	B Cell	Arm-level Gain	0.326898323
GBM	CD8+ T Cell	Arm-level Deletion	0.330259296
GBM	CD8+ T Cell	Diploid/Normal	1
GBM	CD8+ T Cell	Arm-level Gain	0.42245805
GBM	CD4+ T Cell	Arm-level Deletion	0.839398862
GBM	CD4+ T Cell	Diploid/Normal	1
GBM	CD4+ T Cell	Arm-level Gain	0.355302228
GBM	Macrophage	Arm-level Deletion	0.502320465
GBM	Macrophage	Diploid/Normal	1
GBM	Macrophage	Arm-level Gain	0.134774599
GBM	Neutrophil	Arm-level Deletion	0.853324697
GBM	Neutrophil	Diploid/Normal	1
GBM	Neutrophil	Arm-level Gain	0.360292876
GBM	Dendritic Cell	Arm-level Deletion	0.380757039
GBM	Dendritic Cell	Diploid/Normal	1
GBM	Dendritic Cell	Arm-level Gain	0.085291476
HNSC	B Cell	Arm-level Deletion	0.295152932
HNSC	B Cell	Diploid/Normal	1
HNSC	B Cell	Arm-level Gain	0.007004918
HNSC	B Cell	High Amplication	0.00184183
HNSC	CD8+ T Cell	Arm-level Deletion	0.005544454
HNSC	CD8+ T Cell	Diploid/Normal	1

HNSC	CD8+ T Cell	Arm-level Gain	0.010013966
HNSC	CD8+ T Cell	High Amplication	0.000579518
HNSC	CD4+ T Cell	Arm-level Deletion	0.162380733
HNSC	CD4+ T Cell	Diploid/Normal	1
HNSC	CD4+ T Cell	Arm-level Gain	0.174125285
HNSC	CD4+ T Cell	High Amplication	0.209130675
HNSC	Macrophage	Arm-level Deletion	0.34796129
HNSC	Macrophage	Diploid/Normal	1
HNSC	Macrophage	Arm-level Gain	0.42121017
HNSC	Macrophage	High Amplication	0.310806562
HNSC	Neutrophil	Arm-level Deletion	0.005715122
HNSC	Neutrophil	Diploid/Normal	1
HNSC	Neutrophil	Arm-level Gain	0.696824904
HNSC	Neutrophil	High Amplication	0.390504579
HNSC	Dendritic Cell	Arm-level Deletion	0.008820409
HNSC	Dendritic Cell	Diploid/Normal	1
HNSC	Dendritic Cell	Arm-level Gain	0.089163903
HNSC	Dendritic Cell	High Amplication	0.111062386
HNSC-HPVneg	B Cell	Arm-level Deletion	0.913615981
HNSC-HPVneg	B Cell	Diploid/Normal	1
HNSC-HPVneg	B Cell	Arm-level Gain	0.197053737
HNSC-HPVneg	B Cell	High Amplication	0.225902185
HNSC-HPVneg	CD8+ T Cell	Arm-level Deletion	0.094905213
HNSC-HPVneg	CD8+ T Cell	Diploid/Normal	1
HNSC-HPVneg	CD8+ T Cell	Arm-level Gain	0.141524947
HNSC-HPVneg	CD8+ T Cell	High Amplication	0.057219535
HNSC-HPVneg	CD4+ T Cell	Arm-level Deletion	0.312276234
HNSC-HPVneg	CD4+ T Cell	Diploid/Normal	1
HNSC-HPVneg	CD4+ T Cell	Arm-level Gain	0.855608393
HNSC-HPVneg	CD4+ T Cell	High Amplication	0.565765813
HNSC-HPVneg	Macrophage	Arm-level Deletion	0.542102267
HNSC-HPVneg	Macrophage	Diploid/Normal	1
HNSC-HPVneg	Macrophage	Arm-level Gain	0.865527164
HNSC-HPVneg	Macrophage	High Amplication	0.369761092
HNSC-HPVneg	Neutrophil	Arm-level Deletion	0.019143692
HNSC-HPVneg	Neutrophil	Diploid/Normal	1
HNSC-HPVneg	Neutrophil	Arm-level Gain	0.549330117
HNSC-HPVneg	Neutrophil	High Amplication	0.480453832
HNSC-HPVneg	Dendritic Cell	Arm-level Deletion	0.072265549
HNSC-HPVneg	Dendritic Cell	Diploid/Normal	1
HNSC-HPVneg	Dendritic Cell	Arm-level Gain	0.546239622
HNSC-HPVneg	Dendritic Cell	High Amplication	0.443839993
HNSC-HPVpos	B Cell	Arm-level Deletion	0.576977423
HNSC-HPVpos	B Cell	Diploid/Normal	1
HNSC-HPVpos	B Cell	Arm-level Gain	0.138361754
HNSC-HPVpos	B Cell	High Amplication	1
HNSC-HPVpos	CD8+ T Cell	Arm-level Deletion	0.24559246
HNSC-HPVpos	CD8+ T Cell	Diploid/Normal	1
HNSC-HPVpos	CD8+ T Cell	Arm-level Gain	0.185678147
HNSC-HPVpos	CD8+ T Cell	High Amplication	1
HNSC-HPVpos	CD4+ T Cell	Arm-level Deletion	0.885868474
HNSC-HPVpos	CD4+ T Cell	Diploid/Normal	1
HNSC-HPVpos	CD4+ T Cell	Arm-level Gain	0.345996995
HNSC-HPVpos	CD4+ T Cell	High Amplication	1
HNSC-HPVpos	Macrophage	Arm-level Deletion	0.550824639
HNSC-HPVpos	Macrophage	Diploid/Normal	1
HNSC-HPVpos	Macrophage	Arm-level Gain	0.282452886
HNSC-HPVpos	Macrophage	High Amplication	1

HNSC-HPVpos	Neutrophil	Arm-level Deletion	0.458605559
HNSC-HPVpos	Neutrophil	Diploid/Normal	1
HNSC-HPVpos	Neutrophil	Arm-level Gain	0.994953612
HNSC-HPVpos	Neutrophil	High Amplication	1
HNSC-HPVpos	Dendritic Cell	Arm-level Deletion	0.334504078
HNSC-HPVpos	Dendritic Cell	Diploid/Normal	1
HNSC-HPVpos	Dendritic Cell	Arm-level Gain	0.204336156
HNSC-HPVpos	Dendritic Cell	High Amplication	1
KICH	B Cell	Arm-level Deletion	0.178571452
KICH	B Cell	Diploid/Normal	1
KICH	B Cell	Arm-level Gain	0.349292334
KICH	B Cell	High Amplication	1
KICH	CD8+ T Cell	Arm-level Deletion	0.781832315
KICH	CD8+ T Cell	Diploid/Normal	1
KICH	CD8+ T Cell	Arm-level Gain	0.144677768
KICH	CD8+ T Cell	High Amplication	1
KICH	CD4+ T Cell	Arm-level Deletion	0.055585174
KICH	CD4+ T Cell	Diploid/Normal	1
KICH	CD4+ T Cell	Arm-level Gain	0.059236652
KICH	CD4+ T Cell	High Amplication	1
KICH	Macrophage	Arm-level Deletion	0.122013318
KICH	Macrophage	Diploid/Normal	1
KICH	Macrophage	Arm-level Gain	0.916494828
KICH	Macrophage	High Amplication	1
KICH	Neutrophil	Arm-level Deletion	0.855419094
KICH	Neutrophil	Diploid/Normal	1
KICH	Neutrophil	Arm-level Gain	0.426480012
KICH	Neutrophil	High Amplication	1
KICH	Dendritic Cell	Arm-level Deletion	0.001675069
KICH	Dendritic Cell	Diploid/Normal	1
KICH	Dendritic Cell	Arm-level Gain	0.625602738
KICH	Dendritic Cell	High Amplication	1
KIRC	B Cell	Deep Deletion	1
KIRC	B Cell	Arm-level Deletion	0.95770001
KIRC	B Cell	Diploid/Normal	1
KIRC	B Cell	Arm-level Gain	0.868226603
KIRC	B Cell	High Amplication	0.689007716
KIRC	CD8+ T Cell	Deep Deletion	1
KIRC	CD8+ T Cell	Arm-level Deletion	0.876703185
KIRC	CD8+ T Cell	Diploid/Normal	1
KIRC	CD8+ T Cell	Arm-level Gain	0.016409975
KIRC	CD8+ T Cell	High Amplication	0.338675726
KIRC	CD4+ T Cell	Deep Deletion	1
KIRC	CD4+ T Cell	Arm-level Deletion	0.054994542
KIRC	CD4+ T Cell	Diploid/Normal	1
KIRC	CD4+ T Cell	Arm-level Gain	0.231270948
KIRC	CD4+ T Cell	High Amplication	0.792003261
KIRC	Macrophage	Deep Deletion	1
KIRC	Macrophage	Arm-level Deletion	0.948487595
KIRC	Macrophage	Diploid/Normal	1
KIRC	Macrophage	Arm-level Gain	0.775477625
KIRC	Macrophage	High Amplication	0.296995985
KIRC	Neutrophil	Deep Deletion	1
KIRC	Neutrophil	Arm-level Deletion	0.198215032
KIRC	Neutrophil	Diploid/Normal	1
KIRC	Neutrophil	Arm-level Gain	0.648899177
KIRC	Neutrophil	High Amplication	0.031631306
KIRC	Dendritic Cell	Deep Deletion	1

KIRC	Dendritic Cell	Arm-level Deletion	0.800080624
KIRC	Dendritic Cell	Diploid/Normal	1
KIRC	Dendritic Cell	Arm-level Gain	0.549972753
KIRC	Dendritic Cell	High Amplication	0.875353625
KIRP	B Cell	Arm-level Deletion	0.671081257
KIRP	B Cell	Diploid/Normal	1
KIRP	B Cell	Arm-level Gain	0.886600571
KIRP	CD8+ T Cell	Arm-level Deletion	0.754219338
KIRP	CD8+ T Cell	Diploid/Normal	1
KIRP	CD8+ T Cell	Arm-level Gain	0.512873332
KIRP	CD4+ T Cell	Arm-level Deletion	0.402776434
KIRP	CD4+ T Cell	Diploid/Normal	1
KIRP	CD4+ T Cell	Arm-level Gain	0.708430859
KIRP	Macrophage	Arm-level Deletion	0.093701016
KIRP	Macrophage	Diploid/Normal	1
KIRP	Macrophage	Arm-level Gain	0.15262797
KIRP	Neutrophil	Arm-level Deletion	0.477856811
KIRP	Neutrophil	Diploid/Normal	1
KIRP	Neutrophil	Arm-level Gain	0.026722432
KIRP	Dendritic Cell	Arm-level Deletion	0.488233792
KIRP	Dendritic Cell	Diploid/Normal	1
KIRP	Dendritic Cell	Arm-level Gain	0.022992172
LGG	B Cell	Arm-level Deletion	0.113844322
LGG	B Cell	Diploid/Normal	1
LGG	B Cell	Arm-level Gain	0.042063977
LGG	B Cell	High Amplication	1
LGG	CD8+ T Cell	Arm-level Deletion	0.227699848
LGG	CD8+ T Cell	Diploid/Normal	1
LGG	CD8+ T Cell	Arm-level Gain	0.807415863
LGG	CD8+ T Cell	High Amplication	1
LGG	CD4+ T Cell	Arm-level Deletion	0.495014492
LGG	CD4+ T Cell	Diploid/Normal	1
LGG	CD4+ T Cell	Arm-level Gain	0.042913777
LGG	CD4+ T Cell	High Amplication	1
LGG	Macrophage	Arm-level Deletion	0.652961727
LGG	Macrophage	Diploid/Normal	1
LGG	Macrophage	Arm-level Gain	0.162796472
LGG	Macrophage	High Amplication	1
LGG	Neutrophil	Arm-level Deletion	0.266067741
LGG	Neutrophil	Diploid/Normal	1
LGG	Neutrophil	Arm-level Gain	0.23458936
LGG	Neutrophil	High Amplication	1
LGG	Dendritic Cell	Arm-level Deletion	0.160304904
LGG	Dendritic Cell	Diploid/Normal	1
LGG	Dendritic Cell	Arm-level Gain	0.189593543
LGG	Dendritic Cell	High Amplication	1
LIHC	B Cell	Arm-level Deletion	0.680243872
LIHC	B Cell	Diploid/Normal	1
LIHC	B Cell	Arm-level Gain	0.383960416
LIHC	B Cell	High Amplication	0.246925876
LIHC	CD8+ T Cell	Arm-level Deletion	0.59676081
LIHC	CD8+ T Cell	Diploid/Normal	1
LIHC	CD8+ T Cell	Arm-level Gain	0.130085535
LIHC	CD8+ T Cell	High Amplication	0.101961127
LIHC	CD4+ T Cell	Arm-level Deletion	0.762731211
LIHC	CD4+ T Cell	Diploid/Normal	1
LIHC	CD4+ T Cell	Arm-level Gain	0.276649418
LIHC	CD4+ T Cell	High Amplication	0.372360491

LIHC	Macrophage	Arm-level Deletion	0.825210578
LIHC	Macrophage	Diploid/Normal	1
LIHC	Macrophage	Arm-level Gain	0.049643278
LIHC	Macrophage	High Amplication	0.575971225
LIHC	Neutrophil	Arm-level Deletion	0.609917789
LIHC	Neutrophil	Diploid/Normal	1
LIHC	Neutrophil	Arm-level Gain	0.102386494
LIHC	Neutrophil	High Amplication	0.627516228
LIHC	Dendritic Cell	Arm-level Deletion	0.880265554
LIHC	Dendritic Cell	Diploid/Normal	1
LIHC	Dendritic Cell	Arm-level Gain	0.265354506
LIHC	Dendritic Cell	High Amplication	0.480002914
LUAD	B Cell	Arm-level Deletion	0.752853023
LUAD	B Cell	Diploid/Normal	1
LUAD	B Cell	Arm-level Gain	0.015529722
LUAD	B Cell	High Amplication	0.007472054
LUAD	CD8+ T Cell	Arm-level Deletion	0.110335427
LUAD	CD8+ T Cell	Diploid/Normal	1
LUAD	CD8+ T Cell	Arm-level Gain	0.165420792
LUAD	CD8+ T Cell	High Amplication	0.412067286
LUAD	CD4+ T Cell	Arm-level Deletion	0.386332267
LUAD	CD4+ T Cell	Diploid/Normal	1
LUAD	CD4+ T Cell	Arm-level Gain	0.00398294
LUAD	CD4+ T Cell	High Amplication	0.000337632
LUAD	Macrophage	Arm-level Deletion	0.068756089
LUAD	Macrophage	Diploid/Normal	1
LUAD	Macrophage	Arm-level Gain	0.004948267
LUAD	Macrophage	High Amplication	0.003673349
LUAD	Neutrophil	Arm-level Deletion	0.207179103
LUAD	Neutrophil	Diploid/Normal	1
LUAD	Neutrophil	Arm-level Gain	0.008824845
LUAD	Neutrophil	High Amplication	0.0005581
LUAD	Dendritic Cell	Arm-level Deletion	0.723027498
LUAD	Dendritic Cell	Diploid/Normal	1
LUAD	Dendritic Cell	Arm-level Gain	0.001781465
LUAD	Dendritic Cell	High Amplication	4.77E-05
LUSC	B Cell	Deep Deletion	1
LUSC	B Cell	Arm-level Deletion	0.00017037
LUSC	B Cell	Diploid/Normal	1
LUSC	B Cell	Arm-level Gain	0.008040943
LUSC	B Cell	High Amplication	0.081451958
LUSC	CD8+ T Cell	Deep Deletion	1
LUSC	CD8+ T Cell	Arm-level Deletion	0.237116098
LUSC	CD8+ T Cell	Diploid/Normal	1
LUSC	CD8+ T Cell	Arm-level Gain	0.408219296
LUSC	CD8+ T Cell	High Amplication	0.613231273
LUSC	CD4+ T Cell	Deep Deletion	1
LUSC	CD4+ T Cell	Arm-level Deletion	0.000104141
LUSC	CD4+ T Cell	Diploid/Normal	1
LUSC	CD4+ T Cell	Arm-level Gain	0.064058405
LUSC	CD4+ T Cell	High Amplication	0.01144558
LUSC	Macrophage	Deep Deletion	1
LUSC	Macrophage	Arm-level Deletion	0.056946039
LUSC	Macrophage	Diploid/Normal	1
LUSC	Macrophage	Arm-level Gain	0.414464596
LUSC	Macrophage	High Amplication	0.170647712
LUSC	Neutrophil	Deep Deletion	1
LUSC	Neutrophil	Arm-level Deletion	0.060090629

LUSC	Neutrophil	Diploid/Normal	1
LUSC	Neutrophil	Arm-level Gain	0.680956978
LUSC	Neutrophil	High Amplication	0.286188901
LUSC	Dendritic Cell	Deep Deletion	1
LUSC	Dendritic Cell	Arm-level Deletion	0.007237345
LUSC	Dendritic Cell	Diploid/Normal	1
LUSC	Dendritic Cell	Arm-level Gain	0.18483543
LUSC	Dendritic Cell	High Amplication	0.279659943
MESO	B Cell	Deep Deletion	0.806230068
MESO	B Cell	Arm-level Deletion	0.463280008
MESO	B Cell	Diploid/Normal	1
MESO	B Cell	Arm-level Gain	0.465809518
MESO	CD8+ T Cell	Deep Deletion	0.240671446
MESO	CD8+ T Cell	Arm-level Deletion	0.801232987
MESO	CD8+ T Cell	Diploid/Normal	1
MESO	CD8+ T Cell	Arm-level Gain	0.273868392
MESO	CD4+ T Cell	Deep Deletion	0.502883343
MESO	CD4+ T Cell	Arm-level Deletion	0.932933328
MESO	CD4+ T Cell	Diploid/Normal	1
MESO	CD4+ T Cell	Arm-level Gain	0.034693378
MESO	Macrophage	Deep Deletion	0.681369474
MESO	Macrophage	Arm-level Deletion	0.117551332
MESO	Macrophage	Diploid/Normal	1
MESO	Macrophage	Arm-level Gain	0.862736204
MESO	Neutrophil	Deep Deletion	4.23E-06
MESO	Neutrophil	Arm-level Deletion	0.208521871
MESO	Neutrophil	Diploid/Normal	1
MESO	Neutrophil	Arm-level Gain	0.018779916
MESO	Dendritic Cell	Deep Deletion	0.717367864
MESO	Dendritic Cell	Arm-level Deletion	0.307765821
MESO	Dendritic Cell	Diploid/Normal	1
MESO	Dendritic Cell	Arm-level Gain	0.819924429
OV	B Cell	Arm-level Deletion	0.197137744
OV	B Cell	Diploid/Normal	1
OV	B Cell	Arm-level Gain	0.01484951
OV	B Cell	High Amplication	0.11075478
OV	CD8+ T Cell	Arm-level Deletion	0.21468677
OV	CD8+ T Cell	Diploid/Normal	1
OV	CD8+ T Cell	Arm-level Gain	0.844298763
OV	CD8+ T Cell	High Amplication	0.91268083
OV	CD4+ T Cell	Arm-level Deletion	0.568562375
OV	CD4+ T Cell	Diploid/Normal	1
OV	CD4+ T Cell	Arm-level Gain	0.91134183
OV	CD4+ T Cell	High Amplication	0.77439692
OV	Macrophage	Arm-level Deletion	0.484814595
OV	Macrophage	Diploid/Normal	1
OV	Macrophage	Arm-level Gain	0.042760352
OV	Macrophage	High Amplication	0.989064423
OV	Neutrophil	Arm-level Deletion	0.464151621
OV	Neutrophil	Diploid/Normal	1
OV	Neutrophil	Arm-level Gain	0.712527742
OV	Neutrophil	High Amplication	0.830053469
OV	Dendritic Cell	Arm-level Deletion	0.166470215
OV	Dendritic Cell	Diploid/Normal	1
OV	Dendritic Cell	Arm-level Gain	0.552282845
OV	Dendritic Cell	High Amplication	0.468332448
PAAD	B Cell	Arm-level Deletion	0.019191243
PAAD	B Cell	Diploid/Normal	1

PAAD	B Cell	Arm-level Gain	0.031896219
PAAD	B Cell	High Amplication	0.050359578
PAAD	CD8+ T Cell	Arm-level Deletion	0.195022464
PAAD	CD8+ T Cell	Diploid/Normal	1
PAAD	CD8+ T Cell	Arm-level Gain	0.280977842
PAAD	CD8+ T Cell	High Amplication	0.278396709
PAAD	CD4+ T Cell	Arm-level Deletion	0.023690478
PAAD	CD4+ T Cell	Diploid/Normal	1
PAAD	CD4+ T Cell	Arm-level Gain	0.003655699
PAAD	CD4+ T Cell	High Amplication	0.314201187
PAAD	Macrophage	Arm-level Deletion	0.15548009
PAAD	Macrophage	Diploid/Normal	1
PAAD	Macrophage	Arm-level Gain	0.999938312
PAAD	Macrophage	High Amplication	0.800786148
PAAD	Neutrophil	Arm-level Deletion	0.316036847
PAAD	Neutrophil	Diploid/Normal	1
PAAD	Neutrophil	Arm-level Gain	0.505047443
PAAD	Neutrophil	High Amplication	0.825473471
PAAD	Dendritic Cell	Arm-level Deletion	0.360054436
PAAD	Dendritic Cell	Diploid/Normal	1
PAAD	Dendritic Cell	Arm-level Gain	0.705324934
PAAD	Dendritic Cell	High Amplication	0.408740213
PCPG	B Cell	Arm-level Deletion	0.145424965
PCPG	B Cell	Diploid/Normal	1
PCPG	B Cell	Arm-level Gain	0.66617289
PCPG	B Cell	High Amplication	0.7899961
PCPG	CD8+ T Cell	Arm-level Deletion	0.532329139
PCPG	CD8+ T Cell	Diploid/Normal	1
PCPG	CD8+ T Cell	Arm-level Gain	0.733727176
PCPG	CD8+ T Cell	High Amplication	0.932151829
PCPG	CD4+ T Cell	Arm-level Deletion	0.582718537
PCPG	CD4+ T Cell	Diploid/Normal	1
PCPG	CD4+ T Cell	Arm-level Gain	0.801331766
PCPG	CD4+ T Cell	High Amplication	0.056186739
PCPG	Macrophage	Arm-level Deletion	0.706457504
PCPG	Macrophage	Diploid/Normal	1
PCPG	Macrophage	Arm-level Gain	0.751346298
PCPG	Macrophage	High Amplication	0.86265942
PCPG	Neutrophil	Arm-level Deletion	0.233574665
PCPG	Neutrophil	Diploid/Normal	1
PCPG	Neutrophil	Arm-level Gain	0.139227978
PCPG	Neutrophil	High Amplication	0.965131111
PCPG	Dendritic Cell	Arm-level Deletion	0.078461317
PCPG	Dendritic Cell	Diploid/Normal	1
PCPG	Dendritic Cell	Arm-level Gain	0.717799838
PCPG	Dendritic Cell	High Amplication	0.228764367
PRAD	B Cell	Deep Deletion	0.935436882
PRAD	B Cell	Arm-level Deletion	0.606643479
PRAD	B Cell	Diploid/Normal	1
PRAD	B Cell	Arm-level Gain	0.452589927
PRAD	B Cell	High Amplication	0.368802047
PRAD	CD8+ T Cell	Deep Deletion	0.905005255
PRAD	CD8+ T Cell	Arm-level Deletion	0.875775857
PRAD	CD8+ T Cell	Diploid/Normal	1
PRAD	CD8+ T Cell	Arm-level Gain	0.34405176
PRAD	CD8+ T Cell	High Amplication	0.455019107
PRAD	CD4+ T Cell	Deep Deletion	0.444058935
PRAD	CD4+ T Cell	Arm-level Deletion	0.398971486

PRAD	CD4+ T Cell	Diploid/Normal	1
PRAD	CD4+ T Cell	Arm-level Gain	0.87684277
PRAD	CD4+ T Cell	High Amplication	0.11049785
PRAD	Macrophage	Deep Deletion	0.963476011
PRAD	Macrophage	Arm-level Deletion	0.816926028
PRAD	Macrophage	Diploid/Normal	1
PRAD	Macrophage	Arm-level Gain	0.583048098
PRAD	Macrophage	High Amplication	0.000410857
PRAD	Neutrophil	Deep Deletion	0.165272348
PRAD	Neutrophil	Arm-level Deletion	0.95389178
PRAD	Neutrophil	Diploid/Normal	1
PRAD	Neutrophil	Arm-level Gain	0.781817521
PRAD	Neutrophil	High Amplication	0.368121942
PRAD	Dendritic Cell	Deep Deletion	0.607321305
PRAD	Dendritic Cell	Arm-level Deletion	0.89757881
PRAD	Dendritic Cell	Diploid/Normal	1
PRAD	Dendritic Cell	Arm-level Gain	0.912282317
PRAD	Dendritic Cell	High Amplication	0.217911445
READ	B Cell	Arm-level Deletion	0.698480431
READ	B Cell	Diploid/Normal	1
READ	B Cell	Arm-level Gain	0.923086883
READ	B Cell	High Amplication	0.037034391
READ	CD8+ T Cell	Arm-level Deletion	0.182759976
READ	CD8+ T Cell	Diploid/Normal	1
READ	CD8+ T Cell	Arm-level Gain	0.399025869
READ	CD8+ T Cell	High Amplication	0.635806702
READ	CD4+ T Cell	Arm-level Deletion	0.464254727
READ	CD4+ T Cell	Diploid/Normal	1
READ	CD4+ T Cell	Arm-level Gain	0.115160948
READ	CD4+ T Cell	High Amplication	0.542779479
READ	Macrophage	Arm-level Deletion	0.70900053
READ	Macrophage	Diploid/Normal	1
READ	Macrophage	Arm-level Gain	0.04814409
READ	Macrophage	High Amplication	0.1946652
READ	Neutrophil	Arm-level Deletion	0.012008406
READ	Neutrophil	Diploid/Normal	1
READ	Neutrophil	Arm-level Gain	0.299548017
READ	Neutrophil	High Amplication	0.380023762
READ	Dendritic Cell	Arm-level Deletion	0.286723598
READ	Dendritic Cell	Diploid/Normal	1
READ	Dendritic Cell	Arm-level Gain	0.558972724
READ	Dendritic Cell	High Amplication	0.190660011
SARC	B Cell	Deep Deletion	1
SARC	B Cell	Arm-level Deletion	0.017339242
SARC	B Cell	Diploid/Normal	1
SARC	B Cell	Arm-level Gain	0.404513293
SARC	B Cell	High Amplication	0.001343253
SARC	CD8+ T Cell	Deep Deletion	1
SARC	CD8+ T Cell	Arm-level Deletion	0.365493482
SARC	CD8+ T Cell	Diploid/Normal	1
SARC	CD8+ T Cell	Arm-level Gain	0.41399566
SARC	CD8+ T Cell	High Amplication	0.048907311
SARC	CD4+ T Cell	Deep Deletion	1
SARC	CD4+ T Cell	Arm-level Deletion	0.599770826
SARC	CD4+ T Cell	Diploid/Normal	1
SARC	CD4+ T Cell	Arm-level Gain	0.044362154
SARC	CD4+ T Cell	High Amplication	3.13E-05
SARC	Macrophage	Deep Deletion	1

SARC	Macrophage	Arm-level Deletion	0.373115455
SARC	Macrophage	Diploid/Normal	1
SARC	Macrophage	Arm-level Gain	0.177744662
SARC	Macrophage	High Amplication	0.019790142
SARC	Neutrophil	Deep Deletion	1
SARC	Neutrophil	Arm-level Deletion	0.669647241
SARC	Neutrophil	Diploid/Normal	1
SARC	Neutrophil	Arm-level Gain	0.932097846
SARC	Neutrophil	High Amplication	0.163467719
SARC	Dendritic Cell	Deep Deletion	1
SARC	Dendritic Cell	Arm-level Deletion	0.342274087
SARC	Dendritic Cell	Diploid/Normal	1
SARC	Dendritic Cell	Arm-level Gain	0.401786978
SARC	Dendritic Cell	High Amplication	0.012189972
SKCM	B Cell	Arm-level Deletion	0.012235654
SKCM	B Cell	Diploid/Normal	1
SKCM	B Cell	Arm-level Gain	0.003046353
SKCM	B Cell	High Amplication	0.000109137
SKCM	CD8+ T Cell	Arm-level Deletion	7.58E-05
SKCM	CD8+ T Cell	Diploid/Normal	1
SKCM	CD8+ T Cell	Arm-level Gain	0.051057909
SKCM	CD8+ T Cell	High Amplication	0.000459608
SKCM	CD4+ T Cell	Arm-level Deletion	0.05202173
SKCM	CD4+ T Cell	Diploid/Normal	1
SKCM	CD4+ T Cell	Arm-level Gain	0.013273876
SKCM	CD4+ T Cell	High Amplication	8.25E-06
SKCM	Macrophage	Arm-level Deletion	0.175214796
SKCM	Macrophage	Diploid/Normal	1
SKCM	Macrophage	Arm-level Gain	0.485354438
SKCM	Macrophage	High Amplication	0.078055991
SKCM	Neutrophil	Arm-level Deletion	0.014239462
SKCM	Neutrophil	Diploid/Normal	1
SKCM	Neutrophil	Arm-level Gain	0.051088147
SKCM	Neutrophil	High Amplication	0.023861872
SKCM	Dendritic Cell	Arm-level Deletion	0.004098064
SKCM	Dendritic Cell	Diploid/Normal	1
SKCM	Dendritic Cell	Arm-level Gain	0.033009991
SKCM	Dendritic Cell	High Amplication	0.002588712
STAD	B Cell	Deep Deletion	0.5370205
STAD	B Cell	Arm-level Deletion	0.169911327
STAD	B Cell	Diploid/Normal	1
STAD	B Cell	Arm-level Gain	0.199244839
STAD	B Cell	High Amplication	0.994184079
STAD	CD8+ T Cell	Deep Deletion	0.727412246
STAD	CD8+ T Cell	Arm-level Deletion	2.08E-06
STAD	CD8+ T Cell	Diploid/Normal	1
STAD	CD8+ T Cell	Arm-level Gain	0.002163282
STAD	CD8+ T Cell	High Amplication	0.059181468
STAD	CD4+ T Cell	Deep Deletion	0.67008271
STAD	CD4+ T Cell	Arm-level Deletion	0.030671709
STAD	CD4+ T Cell	Diploid/Normal	1
STAD	CD4+ T Cell	Arm-level Gain	0.048778622
STAD	CD4+ T Cell	High Amplication	0.934580058
STAD	Macrophage	Deep Deletion	9.56E-10
STAD	Macrophage	Arm-level Deletion	0.001052413
STAD	Macrophage	Diploid/Normal	1
STAD	Macrophage	Arm-level Gain	0.005147515
STAD	Macrophage	High Amplication	0.081881362

STAD	Neutrophil	Deep Deletion	0.536374847
STAD	Neutrophil	Arm-level Deletion	0.008511961
STAD	Neutrophil	Diploid/Normal	1
STAD	Neutrophil	Arm-level Gain	0.069709365
STAD	Neutrophil	High Amplication	0.793119836
STAD	Dendritic Cell	Deep Deletion	0.30205392
STAD	Dendritic Cell	Arm-level Deletion	0.000157875
STAD	Dendritic Cell	Diploid/Normal	1
STAD	Dendritic Cell	Arm-level Gain	0.00048019
STAD	Dendritic Cell	High Amplication	0.223286946
TGCT	B Cell	Deep Deletion	1
TGCT	B Cell	Arm-level Deletion	0.038019887
TGCT	B Cell	Diploid/Normal	1
TGCT	B Cell	Arm-level Gain	0.659655988
TGCT	B Cell	High Amplication	0.52620487
TGCT	CD8+ T Cell	Deep Deletion	1
TGCT	CD8+ T Cell	Arm-level Deletion	0.890031249
TGCT	CD8+ T Cell	Diploid/Normal	1
TGCT	CD8+ T Cell	Arm-level Gain	0.121751174
TGCT	CD8+ T Cell	High Amplication	0.068743632
TGCT	CD4+ T Cell	Deep Deletion	1
TGCT	CD4+ T Cell	Arm-level Deletion	0.243861494
TGCT	CD4+ T Cell	Diploid/Normal	1
TGCT	CD4+ T Cell	Arm-level Gain	0.94322294
TGCT	CD4+ T Cell	High Amplication	0.708715687
TGCT	Macrophage	Deep Deletion	1
TGCT	Macrophage	Arm-level Deletion	0.543749329
TGCT	Macrophage	Diploid/Normal	1
TGCT	Macrophage	Arm-level Gain	0.93718841
TGCT	Macrophage	High Amplication	0.357580584
TGCT	Neutrophil	Deep Deletion	1
TGCT	Neutrophil	Arm-level Deletion	0.516684596
TGCT	Neutrophil	Diploid/Normal	1
TGCT	Neutrophil	Arm-level Gain	0.122056104
TGCT	Neutrophil	High Amplication	0.578921366
TGCT	Dendritic Cell	Deep Deletion	1
TGCT	Dendritic Cell	Arm-level Deletion	0.295444919
TGCT	Dendritic Cell	Diploid/Normal	1
TGCT	Dendritic Cell	Arm-level Gain	0.91292108
TGCT	Dendritic Cell	High Amplication	0.917011236
THCA	B Cell	Arm-level Deletion	3.92E-10
THCA	B Cell	Diploid/Normal	1
THCA	B Cell	Arm-level Gain	0.243186773
THCA	CD8+ T Cell	Arm-level Deletion	0.06892855
THCA	CD8+ T Cell	Diploid/Normal	1
THCA	CD8+ T Cell	Arm-level Gain	0.107637729
THCA	CD4+ T Cell	Arm-level Deletion	0.001783542
THCA	CD4+ T Cell	Diploid/Normal	1
THCA	CD4+ T Cell	Arm-level Gain	0.751573079
THCA	Macrophage	Arm-level Deletion	0.003495448
THCA	Macrophage	Diploid/Normal	1
THCA	Macrophage	Arm-level Gain	0.386387186
THCA	Neutrophil	Arm-level Deletion	4.68E-06
THCA	Neutrophil	Diploid/Normal	1
THCA	Neutrophil	Arm-level Gain	0.582364507
THCA	Dendritic Cell	Arm-level Deletion	1.11E-07
THCA	Dendritic Cell	Diploid/Normal	1
THCA	Dendritic Cell	Arm-level Gain	0.430055694

THYM	B Cell	Arm-level Deletion	0.973805067
THYM	B Cell	Diploid/Normal	1
THYM	B Cell	Arm-level Gain	0.519471546
THYM	CD8+ T Cell	Arm-level Deletion	0.67866728
THYM	CD8+ T Cell	Diploid/Normal	1
THYM	CD8+ T Cell	Arm-level Gain	0.424833321
THYM	CD4+ T Cell	Arm-level Deletion	0.026908438
THYM	CD4+ T Cell	Diploid/Normal	1
THYM	CD4+ T Cell	Arm-level Gain	0.169393374
THYM	Macrophage	Arm-level Deletion	0.489841968
THYM	Macrophage	Diploid/Normal	1
THYM	Macrophage	Arm-level Gain	0.721679781
THYM	Neutrophil	Arm-level Deletion	0.832343404
THYM	Neutrophil	Diploid/Normal	1
THYM	Neutrophil	Arm-level Gain	0.341259453
THYM	Dendritic Cell	Arm-level Deletion	0.094709146
THYM	Dendritic Cell	Diploid/Normal	1
THYM	Dendritic Cell	Arm-level Gain	0.072151567
UCEC	B Cell	Arm-level Deletion	0.568903446
UCEC	B Cell	Diploid/Normal	1
UCEC	B Cell	Arm-level Gain	0.006265626
UCEC	B Cell	High Amplication	0.716482556
UCEC	CD8+ T Cell	Arm-level Deletion	0.619596929
UCEC	CD8+ T Cell	Diploid/Normal	1
UCEC	CD8+ T Cell	Arm-level Gain	0.002038957
UCEC	CD8+ T Cell	High Amplication	0.881089856
UCEC	CD4+ T Cell	Arm-level Deletion	0.077630428
UCEC	CD4+ T Cell	Diploid/Normal	1
UCEC	CD4+ T Cell	Arm-level Gain	0.032962086
UCEC	CD4+ T Cell	High Amplication	0.688677026
UCEC	Macrophage	Arm-level Deletion	0.81097893
UCEC	Macrophage	Diploid/Normal	1
UCEC	Macrophage	Arm-level Gain	0.407295301
UCEC	Macrophage	High Amplication	0.556433494
UCEC	Neutrophil	Arm-level Deletion	0.611383465
UCEC	Neutrophil	Diploid/Normal	1
UCEC	Neutrophil	Arm-level Gain	0.60005052
UCEC	Neutrophil	High Amplication	0.339276082
UCEC	Dendritic Cell	Arm-level Deletion	0.863856978
UCEC	Dendritic Cell	Diploid/Normal	1
UCEC	Dendritic Cell	Arm-level Gain	1.11E-06
UCEC	Dendritic Cell	High Amplication	0.992753126
UCS	B Cell	Deep Deletion	1
UCS	B Cell	Arm-level Deletion	0.354824385
UCS	B Cell	Diploid/Normal	1
UCS	B Cell	Arm-level Gain	0.638711847
UCS	B Cell	High Amplication	0.063461489
UCS	CD8+ T Cell	Deep Deletion	1
UCS	CD8+ T Cell	Arm-level Deletion	0.798312241
UCS	CD8+ T Cell	Diploid/Normal	1
UCS	CD8+ T Cell	Arm-level Gain	0.569796647
UCS	CD8+ T Cell	High Amplication	0.887350479
UCS	CD4+ T Cell	Deep Deletion	1
UCS	CD4+ T Cell	Arm-level Deletion	0.21956129
UCS	CD4+ T Cell	Diploid/Normal	1
UCS	CD4+ T Cell	Arm-level Gain	0.252750807
UCS	CD4+ T Cell	High Amplication	0.990333667
UCS	Macrophage	Deep Deletion	1

UCS	Macrophage	Arm-level Deletion	0.531820317
UCS	Macrophage	Diploid/Normal	1
UCS	Macrophage	Arm-level Gain	0.407797462
UCS	Macrophage	High Amplication	0.419332262
UCS	Neutrophil	Deep Deletion	1
UCS	Neutrophil	Arm-level Deletion	0.653766566
UCS	Neutrophil	Diploid/Normal	1
UCS	Neutrophil	Arm-level Gain	0.826542838
UCS	Neutrophil	High Amplication	0.367612595
UCS	Dendritic Cell	Deep Deletion	1
UCS	Dendritic Cell	Arm-level Deletion	0.481077852
UCS	Dendritic Cell	Diploid/Normal	1
UCS	Dendritic Cell	Arm-level Gain	0.155568439
UCS	Dendritic Cell	High Amplication	0.40528169
UVM	B Cell	Diploid/Normal	1
UVM	B Cell	Arm-level Gain	0.061400366
UVM	B Cell	High Amplication	0.22443017
UVM	CD8+ T Cell	Diploid/Normal	1
UVM	CD8+ T Cell	Arm-level Gain	0.029013603
UVM	CD8+ T Cell	High Amplication	0.313574185
UVM	CD4+ T Cell	Diploid/Normal	1
UVM	CD4+ T Cell	Arm-level Gain	0.207662592
UVM	CD4+ T Cell	High Amplication	0.39606942
UVM	Macrophage	Diploid/Normal	1
UVM	Macrophage	Arm-level Gain	0.763661802
UVM	Macrophage	High Amplication	0.006211851
UVM	Neutrophil	Diploid/Normal	1
UVM	Neutrophil	Arm-level Gain	0.205202309
UVM	Neutrophil	High Amplication	0.284309407
UVM	Dendritic Cell	Diploid/Normal	1
UVM	Dendritic Cell	Arm-level Gain	0.987421229
UVM	Dendritic Cell	High Amplication	0.533172034
